# Environmental Regulation, Molecular Profiling, and Preliminary Functional Evaluation of Extracellular Vesicles from *Pleurotus tuber-regium*

**DOI:** 10.3390/foods15081439

**Published:** 2026-04-21

**Authors:** Wen Li, Junyi Fang, Xiaoyan Zhang, Mengmeng Xu, Peter Chi Keung Cheung, Guiyang Shi, Lei Chen, Zhongyang Ding

**Affiliations:** 1 Key Laboratory of Carbohydrate Chemistry and Biotechnology of Ministry of Education, School of Biotechnology, Jiangnan University, Wuxi 214122, China; liwenllww@163.com (W.L.);; 2 National Engineering Research Center of Cereal Fermentation and Food Biomanufacturing, Jiangnan University, Wuxi 214122, China; 3 School of Life Sciences, The Chinese University of Hong Kong, Hong Kong 999077, China

**Keywords:** extracellular vesicles, edible mushroom, *Pleurotus tuber-regium*, environmental modulation, preliminary nanocarrier evaluation

## Abstract

Extracellular vesicles (EVs) from the edible mushroom *Pleurotus tuber-regium* (PTR) were investigated with respect to their environmental responsiveness, molecular features, and preliminary functional properties. PTR-EVs were characterized by dynamic light scattering, nanoparticle tracking analysis, and transmission electron microscopy. Proteomic analysis revealed enrichment of ribosomal and proteasomal proteins, redox-related enzymes, and vesicle trafficking components, suggesting non-random molecular representation. Small RNA sequencing identified abundant novel miRNAs with predicted targets involved in nitrogen metabolism, cell wall remodeling, redox regulation, and ubiquitin-mediated proteolysis. Among the tested factors, temperature showed the strongest association with vesicle production, with particle concentration increasing from 1.22 × 10^9^ to 7.31 × 10^9^ particles/mL at 34 °C, approximately six-fold higher than at 30 °C. Transcriptomic profiling showed coordinated repression of cell wall-associated genes and redox enzymes, together with induction of endoplasmic reticulum proteostasis pathways, consistent with stress-associated changes in the cellular context of vesicle release. Ultrasonicated PTR-EVs exhibited enhanced DPPH and ABTS radical-scavenging activities in chemical assays, with DPPH increasing from 59.52% to 71.73% and ABTS from 38.25% to 40.51%. Encapsulation efficiencies reached 32.67% ± 1.3% for proanthocyanidins and 46.01% ± 0.5% for curcumin. PTR-EVs showed the best short-term stability at pH 7 and 4 °C, supporting their further evaluation as an edible fungal vesicle platform for food-related nanoscale delivery.

## 1. Introduction

Extracellular vesicles (EVs) are endogenous nanoscale carriers that transport proteins, nucleic acids, lipids, and small metabolites, and are increasingly being explored for drug delivery and food-related applications [1]. However, most EV studies remain centered on mammalian systems, while food-compatible and fermentation-based EV sources are still insufficiently investigated. Fungal extracellular vesicles constitute a distinct EV category with potential relevance to food applications, because of their controlled cultivation, reduced pathogen-related concerns, and sustainable production [2]. The rigid fungal cell wall and specialized secretory architecture also suggest that fungal EV biogenesis and cargo features may differ from those of mammalian systems. Despite these features, current understanding of fungal EVs remains limited and is derived largely from pathogenic or model organisms such as *Cryptococcus neoformans* [3], *Candida albicans* [4], and *Saccharomyces cerevisiae* [5].

Within the broader fungal kingdom, EVs derived from edible mushrooms represent a particularly relevant yet underexplored subgroup. Edible fungi are widely consumed, generally regarded as safe, and extensively used in food fermentation and functional ingredient development [6]. EVs from edible mushrooms therefore have inherent advantages for food-related applications. Nevertheless, existing studies on fungal EVs rarely distinguish between pathogenic, model, and edible species [7], and systematic investigations focusing specifically on edible mushroom EVs remain scarce. This gap limits both biological understanding and application-oriented evaluation of fungal EVs in food systems.

Existing studies on fungal EVs have mainly focused on vesicle isolation and basic characterization [8]. Recent studies from the past three years have begun to move beyond this descriptive stage. For example, transcriptomic analyses in *C. neoformans* have revealed stress-responsive EV cargo remodeling under host-mimicking conditions [9], while proteomic profiling of *C. albicans* EVs has identified immunomodulatory proteins associated with pathogenesis [10]. In edible fungi, a pioneering study characterized the physicochemical properties of *Ganoderma lucidum* EVs and demonstrated their anti-inflammatory activity in vitro [11]. Comparative studies across fungal species have also suggested that cell wall integrity influences vesicle release [12]. Despite these advances, most available studies still focus on single omics layers or isolated functional observations. Systematic investigations that integrate environmental regulation, EV production, molecular composition, and functional performance within a single framework remain scarce, particularly in edible mushrooms. This gap limits both mechanistic understanding of fungal EV biology and evaluation of edible fungal EVs for safe, sustainable, and food-compatible nanocarrier applications. Environmental factors such as temperature, nutrient availability, and redox status are known to regulate fungal growth, cell wall remodeling, and intracellular trafficking [13]. However, how these cues reshape EV production and cargo features at the molecular level remains poorly defined, and it is still unclear whether increased EV release under stress reflects an active adaptive response or passive membrane disruption.

Functional characterization of fungal EVs presents additional challenges. In plant-derived EVs, antioxidant activity is often attributed to encapsulated secondary metabolites [14]. By contrast, fungal EVs have also been reported to contain endogenous redox-related proteins, suggesting that their molecular composition and functional basis may differ from those of plant EVs. However, whether these molecular features translate into measurable antioxidant activity, and whether edible fungal EVs can serve as carriers for both hydrophilic and hydrophobic compounds, has not been systematically examined. In addition, the stability of these EV preparations under food-relevant pH and temperature conditions remains insufficiently understood.

A major challenge in fungal EV research, especially in higher fungi, is the lack of broadly recognized markers, which complicates EV identification and functional interpretation [15]. Within this context, *Pleurotus tuber-regium* (PTR) was selected as a representative edible mushroom. This species is widely consumed and has been reported to exhibit antioxidant, immunomodulatory, and anti-inflammatory activities at the organism and extract levels [16]. However, its extracellular vesicles have not been systematically studied. Investigating PTR-derived EVs therefore provides an opportunity to examine EV production, environmental responsiveness, molecular composition, and preliminary functional properties in a food-relevant fungal system.

In the present study, extracellular vesicles from *P. tuber-regium* (PTR-EVs) were investigated through an integrated framework encompassing isolation comparison, analysis of EV production under different environmental conditions, molecular characterization, and preliminary functional evaluation. EVs from multiple edible mushrooms were first compared to identify a suitable model system. To address the lack of established markers in higher fungi, proteomic and small RNA profiling were used to characterize the molecular composition of PTR-EVs. Temperature-associated environmental regulation, was then examined in relation to EV yield and transcriptional responses. In addition, the antioxidant activity of PTR-EVs and their capacity to associate with representative hydrophilic and hydrophobic bioactive compounds were evaluated together with their stability under different environmental conditions.

The main novelty of this study lies in combining environmental regulation analysis with integrated molecular characterization of EVs in an edible mushroom system. Particular emphasis was placed on the relationship between temperature-associated changes in EV production and the corresponding transcriptional responses related to cell wall remodeling and proteostasis, as well as on multi-omics profiling of EV cargo through proteomic and small RNA analyses. Based on this framework, the working hypothesis of this study was that temperature stress would be associated with increased PTR-EV production, accompanied by transcriptional changes related to cell wall remodeling and proteostasis, and that the resulting vesicle preparations would show measurable antioxidant activity and cargo-loading potential. Although the functional assays suggest possible food applications, the primary contribution of this work is to provide an integrated framework for investigating environmental responsiveness and molecular features of EVs in edible mushrooms.

## 2. Materials and Methods

### 2.1. Materials

The strains of *Flammulina velutipes*, *Schizophyllum commune*, *Ganoderma lucidum*, *Cordyceps militaris* and *P. tuber-regium* were maintained in our laboratory. Lywallzyme was obtained from the Guangdong Institute of Microbiology (Guangzhou, China). Yeast extract powder was supplied by Angle Yeast Co., Ltd. (Yichang, China). H_2_O_2_ was purchased from Shanghai Vokai Biotechnology Co., Ltd. (Shanghai, China). Curcumin was obtained from J&K Scientific (Beijing, China) Co., Ltd. Glucose, KH_2_PO_4_, MgSO_4_·7H_2_O, Triton X-100, salicylic acid, FeSO_4_·7H_2_O, and other analytical-grade reagents were purchased from Sinopharm Chemical Reagent Co., Ltd. (Shanghai, China).

### 2.2. Culture Media

Fermentation media were formulated as follows (g/L): *F. velutipes*—soybean powder 40, soluble starch 20, KH_2_PO_4_ 2, MgSO_4_·7H_2_O 1; *S. commune*—glucose 40, peptone 10, yeast extract 5, KH_2_PO_4_ 1, MgSO_4_·7H_2_O 1, VB 0.1; *G. lucidum*—glucose 20, peptone 5, YNB 5, KH_2_PO_4_ 4.5, MgSO_4_·7H_2_O 2; *C. militaris*—glucose 16.4, yeast extract 5, KNO_3_ 1, MgSO_4_ 0.2, KH_2_PO_4_ 1.8, FeSO_4_ 0.02; *P. tuber-regium*—glucose 33, yeast extract 4, KH_2_PO_4_ 1, MgSO_4_·7H_2_O 0.6.

### 2.3. Comparison of EV Yield Among Edible Mushrooms

#### 2.3.1. Isolation from Fermentation Broths

Liquid seed cultures of *F. velutipes*, *S. commune*, *G. lucidum*, *C. militaris*, and *P. tuber-regium* were inoculated at 5% (*v*/*v*) into 100 mL of their respective media. Cultivation was performed under species-specific conditions: *F. velutipes* (25 °C, 160 r/min) [17], *S. commune* (28 °C, 160 r/min) [18], *G. lucidum* (30 °C, 150 r/min) [19], *C. militaris* (22 °C, 140 r/min) [20], and *P. tuber-regium* (30 °C, 180 r/min) [21]. After 7 d, fermentation broths were filtered through a 300-mesh nylon filter to remove mycelia. The filtrates were centrifuged at 10,000× *g* for 30 min at 4 °C (CF16RX II, Hitachi, Ltd., Hitachi, Japan) to remove residual debris [22]. The resulting supernatants were then subjected to differential ultracentrifugation at 40,000× *g* for 90 min at 4 °C using an ultrafast cryo-centrifuge (CP80NX with P32ST rotor, Eppendorf, Hamburg, Germany) [23]. EV pellets were resuspended in 1 mL of 0.22 μm-filtered phosphate-buffered saline (PBS) and stored at 4 °C for up to 4 days prior to analysis [24].

#### 2.3.2. Particle Characterization

EV suspensions were first characterized by dynamic light scattering (DLS). Samples (1 mL) were filtered through a 0.45 μm PES membrane and determined using a nanoparticle size and zeta potential analyzer (ZEN3700, Malvern Panalytical, Malvern, UK). Measurements were conducted at 25 °C with a scattering angle of 173° and a laser wavelength of 633 nm. The refractive index (n) of the dispersant (water) was set to 1.330. The scattering vector (k) was calculated as 0.0263 nm^−1^ using the standard formula $\kappa =\frac{4\pi n}{\lambda }\sin\frac{\theta }{2}$. Results were expressed as the mean of three replicates [25]. Particle size distribution and concentration were further measured by nanoparticle tracking analysis (NTA). EV samples were diluted with 1× PBS, filtered through a 0.45 μm syringe filter, and evaluated using a Nanosight NS300 system (Malvern Panalytical, Malvern, UK) equipped with a 488 nm laser and sCMOS camera. Videos were recorded for 30 s (749 frames at 25 fps) with the camera level set to 12, slider shutter set to 1200, and slider gain set to 125. The detection threshold was set to 15, with blur size and max jump distance set to auto. Measurements were performed at 25 °C, with water viscosity set to 1.034–1.036 cP, and data were processed using the corresponding analysis software (v3.4) [26].

### 2.4. Isolation of PTR-EV

*P. tuber-regium* was selected for subsequent experiments based on its higher EV concentration and narrower size distribution. PTR-EVs were isolated from fermentation broth and enzymatic digests using differential centrifugation or sequential membrane filtration.

#### 2.4.1. Isolation from Fermentation Broth

Differential centrifugation was performed as described in Section 2.3.1. For sequential membrane filtration, clarified fermentation supernatants were passed through 0.8, 0.45, 0.22, and 0.1 μm membranes in sequence. After filtration, each membrane was rinsed with 1 mL of 0.22 μm-filtered 1× PBS, and the eluates were collected separately for further analysis [22].

#### 2.4.2. Isolation from Enzymatic Digest

Fermentation broth was first filtered through a sterile 300-mesh nylon cloth to collect mycelia, which were washed 2–3 times with 0.6 M sucrose. Lywallzyme was dissolved in 0.6 M sucrose at a final concentration of 10 mg/mL and sterilized by filtration through a 0.22 μm membrane. The enzyme solution was mixed with the washed mycelia and incubated at 30 °C with shaking at 150 r/min for 5–6 h. After digestion, the mixture was filtered through a sterile 40 μm cell strainer to remove protoplasts and large debris [27]. The filtrate was centrifuged at 10,000× *g* for 30 min at 4 °C to remove residual fragments. The resulting supernatant was further centrifuged at 100,000× *g* for 90 min at 4 °C, and the pellets were resuspended in 1 mL of sterile 1× PBS. For sequential membrane filtration, the clarified digest was processed using the same membrane series (0.8–0.1 μm), followed by PBS elution from each membrane [28].

### 2.5. Identification of PTR-EVs

#### 2.5.1. Morphological Analysis by Transmission Electron Microscopy (TEM)

Considering the isolation efficiency, subsequent identification and omics analyses were performed using EVs prepared by differential centrifugation of the fermentation broth. PTR-EV suspensions were deposited onto Carbon-coated Formvar Film (3.05 mm, 350 mesh; Zhongjingkeyi (Beijing) Film Technology Co., Ltd., Beijing, China), negatively stained with 2% uranyl acetate, air-dried at room temperature, and imaged using TEM (H-7650, Hitachi, Ltd., Hitachi, Japan) [29]. Vesicle diameter and size distribution were quantified from TEM images using ImageJ software (v2.3.0/1.53q).

#### 2.5.2. Proteomic and Small RNA Analyses

PTR-EV protein concentration was determined by bicinchoninic acid (BCA) assay and sample quality was checked via SDS-PAGE. Proteins were denatured, reduced/alkylated, digested, and peptides were analyzed by nanoLC–MS/MS (DIA) using a nano-UPLC (Vanquish Neo, Thermo Fisher Scientific, Waltham, MA, USA) coupled to an Astral mass spectrometer (Astral mass spectrometer, Thermo Fisher Scientific, Waltham, MA, USA) with a nano-electrospray source and a reversed-phase EASY-Spray column (150 μm × 15 cm) [30]. Small RNA libraries were prepared from 1 μg total RNA or 10 ng small RNA using 3′/5′ adapter ligation, reverse transcription, PCR amplification, gel-based size selection, and sequencing (PE150) [31]. Reads were trimmed and filtered, miRNAs were identified with miRDeep-P2, and targets were predicted with TargetFinder (v1.7). Detailed procedures, instrument parameters, and bioinformatic settings are provided in the Supplementary Materials.

### 2.6. Factors Affecting PTR-EVs Production

EV concentration determined by NTA was used as the primary response variable. A one-factor-at-a-time design was applied based on a basal medium containing glucose (33 g/L), yeast extract (4 g/L), KH_2_PO_4_ (1 g/L), and MgSO_4_·7H_2_O (0.6 g/L), under standard conditions (30 °C, 180 r/min). Individual parameters were varied independently, including fermentation time (2–7 d), temperature (26–34 °C), agitation speed (140–220 r/min), initial pH (4.0–8.0), yeast extract concentration (1–5 g/L), and H_2_O_2_ supplementation (0.4–2.0 mmol/L). Biomass was measured as dry weight after lyophilization. This design was intended as a preliminary screening strategy to identify major environmental factors associated with PTR-EV production, rather than as a full multivariate optimization model.

### 2.7. RNA-Seq Analysis of Temperature-Responsive PTR

*P. tuber-regium* was cultured for 6 d at 30 °C (control) or 34 °C (temperature treatment), with three biological replicates per condition. Mycelia were harvested, washed with sterile PBS, snap-frozen in liquid nitrogen, and stored at −80 °C for less than 24 h prior to RNA extraction. RNA sequencing and library construction were conducted by Gene Denovo Co., Ltd. (Guangzhou, China). Differentially expressed genes (DEGs) were identified using a threshold of FDR < 0.05 and |log_2_FC| ≥ 1. RT-qPCR validation was performed using primers designed with the Primer-BLAST tool (https://www.ncbi.nlm.nih.gov/tools/primer-blast/; accessed on 7 December 2025). Primer sequences for the selected genes are listed in Table S1. RT-qPCR was carried out using ChamQ^®^ Universal SYBR qPCR Master Mix (Vazyme Biotech, Nanjing, China).

### 2.8. Evaluation on Antioxidant Activity of PTR-EV

#### 2.8.1. DPPH Radical Scavenging Assay

PTR-EVs were prepared as three groups following Li et al. [32]: intact EVs (Sample A), ultrasonicated EVs (Sample B), and Triton X-100–treated EVs (Sample C). Sample A was obtained by resuspending EV pellets in 1× PBS. Sample B was prepared by ultrasonication (JY92-IIDN, Scientz Biotechnology Co., Ltd., Ningbo, China) at 60% power of 900 W using 2 s on/2 s off pulses for 1 min. Sample C was prepared by incubating EVs with 0.2% Triton X-100 at room temperature for 30 min. Ultrasonication efficiency was preliminarily evaluated at 20%, 40%, 60%, and 80% power by measuring nucleic acid release at 260 nm (Q5000, Quawell Technology, Inc., San Jose, CA, USA), with intact EVs used as the control.

For the DPPH assay, 100 μL of sample was mixed with 100 μL of 2 × 10^−4^ mol/L DPPH solution, incubated in the dark for 20 min, and measured at 517 nm using a microplate reader (Spark, Tecan Trading Co., Ltd., Shanghai, China). Scavenging activity was calculated as:

(1) $$Y=\left(1-\frac{A_{i}-A_{j}}{A_{c}}\right)\times 100\%,$$

where *A_j_* is the absorbance of the sample with DPPH, *A_j_* is the absorbance of the sample with ethanol, and *A_c_* is the absorbance of solvent with DPPH. Vitamin C (0.1 mg/mL) served as a positive control.

#### 2.8.2. ABTS Radical Scavenging Assay

ABTS•^+^ was generated by mixing 2 mM ABTS with K_2_S_2_O_8_ (1:1, *v*/*v*) and incubating the mixture in the dark for 12–16 h. The working solution was diluted with PBS or ethanol to an absorbance of 0.70 ± 0.02 at 734 nm. Samples (20 μL) were mixed with 180 μL of ABTS working solution, incubated for 6 min, and measured at 734 nm. Scavenging activity was calculated using Equation (1). Vitamin C (0.1 mg/mL) was used as a positive control [33].

### 2.9. Encapsulation and Stability of PTR-EVs

#### 2.9.1. Encapsulation Efficiency of Proanthocyanidins and Curcumin

Proanthocyanidins were dissolved in 0.1 M PBS, and curcumin was dissolved in anhydrous ethanol. Standard curves were generated at 280 nm for proanthocyanidins (0–0.5 mg/mL) [34] and 420 nm for curcumin (0.05–10 μg/mL) [35]. The calibration equations were y = 2.9166x + 0.091 (R^2^ = 0.995) for proanthocyanidins and y = 0.062x + 0.0034 (R^2^ = 0.998) for curcumin, where x is the concentration and y is the absorbance.

For cargo loading, proanthocyanidin solution (300 μL, 3 mg/mL) or curcumin solution (70 μL, 0.1 mg/mL) was added to 1 mL of EV suspension. Encapsulation was performed following Kumar et al. [36] using three approaches: (i) co-incubation at 30 °C and 150 r/min for 2 h (Sample 1); (ii) ultrasonication at 20% power (180 W) with 3 s on/3 s off pulses for 10 cycles and 2 min cooling intervals, followed by equilibration at room temperature for 1 h (Sample 2); and (iii) Triton X-100 permeabilization (0.2%) for 30 min prior to cargo addition, followed by shaking at 30 °C and 150 r/min for 2 h (Sample 3).

All samples were centrifuged in 100 kDa ultrafiltration tubes at 8000× *g* for 10 min at 4 °C, and free cargo in the filtrate was quantified spectrophotometrically. Encapsulation efficiency (EE) was calculated using Equation (2) as follows:

(2) $$\mathrm{EE}=\frac{W_{t}-W_{d}}{W_{t}}\times 100\%$$

where *W_t_* is the total added cargo and *W_d_* is the free cargo (unencapsulated).

#### 2.9.2. Stability Assessment of Encapsulated PTR-EVs

Stability was evaluated using encapsulated EVs prepared by the most efficient loading method for each cargo. For pH stability, samples were adjusted to pH 3, 5, 7, 9, or 11 using HCl or NaOH and monitored over 72 h at room temperature. For thermal stability, samples were stored at −80, −20, 4, 20, and 30 °C and monitored over 72 h. Transmittance at 600 nm was recorded over time, and particle size and polydispersity index (PDI) were measured after 24 h and 72 h. The colloidal stability of PTR-EVs was assessed using a turbidity-based assay, adapted from methods used for liposome stability evaluation [37].

### 2.10. Data Processing and Statistical Analysis

For EV isolation and characterization, three independent culture batches of PTR were prepared as biological replicates. For each biological replicate, DLS, NTA, antioxidant related assays, and stability-related measurements were performed in triplicate as technical replicates where applicable. TEM imaging was conducted using samples from each biological replicate. Proteomic analysis, small RNA analysis, transcriptomic analysis, and loading experiments were performed using samples from the three biological replicates. Data processing and visualization were carried out using OriginPro 2025. Statistical significance was evaluated by one-way ANOVA followed by Tukey’s post hoc test in SPSS (v25.0), with *p* < 0.05 considered significant. ANOVA results are reported with *F*-values, degrees of freedom, exact *p*-values, and effect sizes where appropriate.

## 3. Results

### 3.1. Comparison of EV Yield Among Selected Edible Mushrooms

DLS and NTA analyses revealed marked differences in EV size distribution and particle yield among the tested mushrooms (Figure S1, Table S2). *P. tuber-regium* produced EVs with a relatively narrow size range (30–650 nm), strong overlap among DLS profiles, and the highest particle concentration reaching (112.0 ± 4.2) × 10^7^ particles/mL. *G. lucidum* also showed a relatively uniform size distribution (80–550 nm) but its EV concentration was much lower at (13.0 ± 0.7) × 10^7^ particles/mL. *F. velutipes* yielded a higher particle concentration of (46.5 ± 1.9) × 10^7^ particles/mL but with a much broader size distribution (60–2000 nm). *C. militaris* produced EVs with the broadest size distribution (60–3000 nm), weak profile overlap, and a relatively low concentration of (8.9 ± 0.4) × 10^7^ particles/mL. *S. commune* showed the lowest EV yield, with a particle concentration of only (2.0 ± 0.2) × 10^7^ particles/mL and a highly heterogeneous size distribution (200–1000 nm). Considering both particle yield and size consistency, *P. tuber-regium* was selected for further investigation.

### 3.2. Performance of PTR-EV Isolation Methods

**Figure 1:**
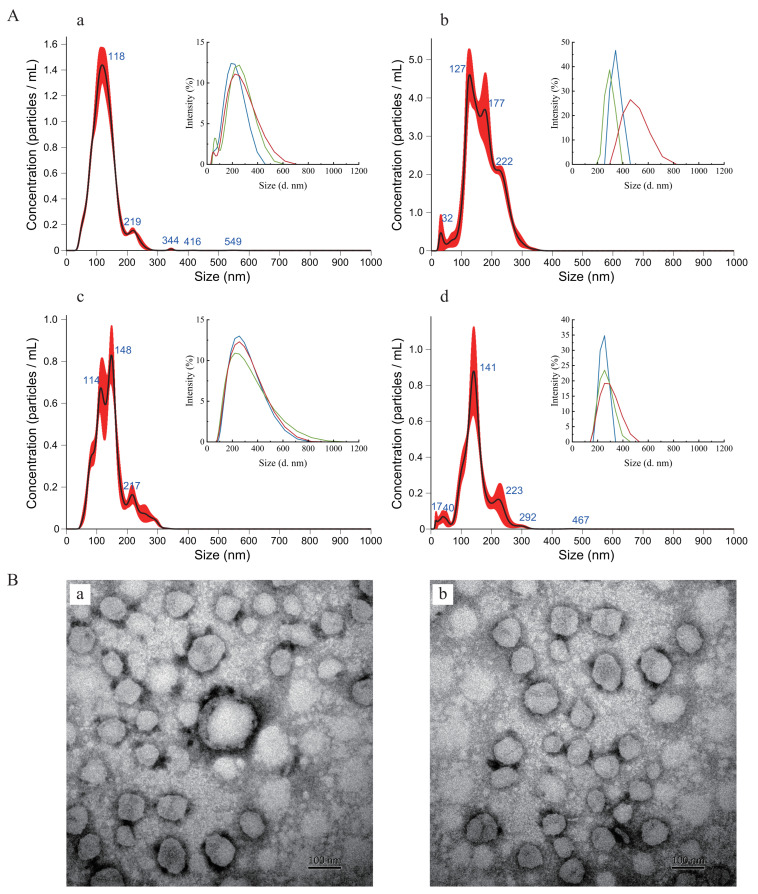
Characteristics of PTR-EVs isolated by different methods. (**A**) NTA and DLS profiles of PTR-EVs isolated by differential centrifugation or ultrafiltration from fermentation broth or enzymatic digest. Insets show corresponding DLS plots. Panels are arranged as follows: the first row (**a**,**b**) represents samples from fermentation broth, and the second row (**c**,**d**) represents samples from enzymatic digest; the first column (**a**,**c**) shows results obtained by differential centrifugation, and the second column (**b**,**d**) shows results obtained by ultrafiltration. The red curve shows the measured particle size distribution, the black curve shows the fitted distribution, and the blue values indicate the characteristic peak sizes (nm). In the inset, the blue, green, and red curves show the size distributions from independent replicate measurements. (**B**) Representative TEM images (**a**,**b**) of PTR-EVs isolated from the fermentation broth by centrifugation. Scale bar: 100 nm.

PTR-EVs were isolated from both fermentation broth and enzymatic digests using either differential centrifugation or sequential membrane filtration. Among the tested methods, differential centrifugation of fermentation broth produced EVs with the most concentrated size distribution and the highest particle yield. DLS analysis showed a size range of 30–650 nm and an average diameter of 196.4 ± 19.3 nm, with a single dominant peak and strong overlap among replicate curves (Figure 1A). NTA further confirmed the highest particle concentration, reaching (12.3 ± 0.2) × 10^8^ particles/mL (Table 1, Figure 1). The corresponding NTA mean and mode particle sizes were 125.0 and 115.3 nm, respectively, consistent with a relatively concentrated size distribution. In contrast, sequential membrane filtration of fermentation broth generated larger and more heterogeneous particles, with a size range of 200–820 nm, an average diameter of 371.2 ± 99.0 nm, weak overlap among DLS curves, and the lowest particle concentration of (5.2 ± 0.4) × 10^8^ particles/mL.

Differential centrifugation of enzymatic digest produced EVs with a broader size range (70–1100 nm), an average diameter of 247.8 ± 3.8 nm, and an intermediate particle concentration of (7.2 ± 0.7) × 10^8^ particles/mL. Sequential membrane filtration of enzymatic digest yielded particles ranging from 140 to 500 nm, with an average diameter of 260.5 ± 18.3 nm, weak profile overlap, and a particle concentration of (6.3 ± 0.7) × 10^8^ particles/mL. Overall, DLS profiles indicated that most PTR-EVs were distributed within 50–600 nm (Figure 1A). Because differential centrifugation of fermentation broth produced preparations with a concentrated size distribution, a dominant DLS peak, strong profile overlap, and the highest particle concentration in both DLS and NTA analyses, all subsequent characterization and experiments were performed using EVs prepared by this method. TEM analysis of these preparations further showed spherical vesicle-like particles with diameters ranging from 48.9 to 158.2 nm and a mean diameter of 78.8 ± 15.2 nm (Figure 1B).

### 3.3. Proteomic Analysis of PTR-EVs

#### 3.3.1. GO Annotation of the PTR-EV Proteome

**Figure 2:**
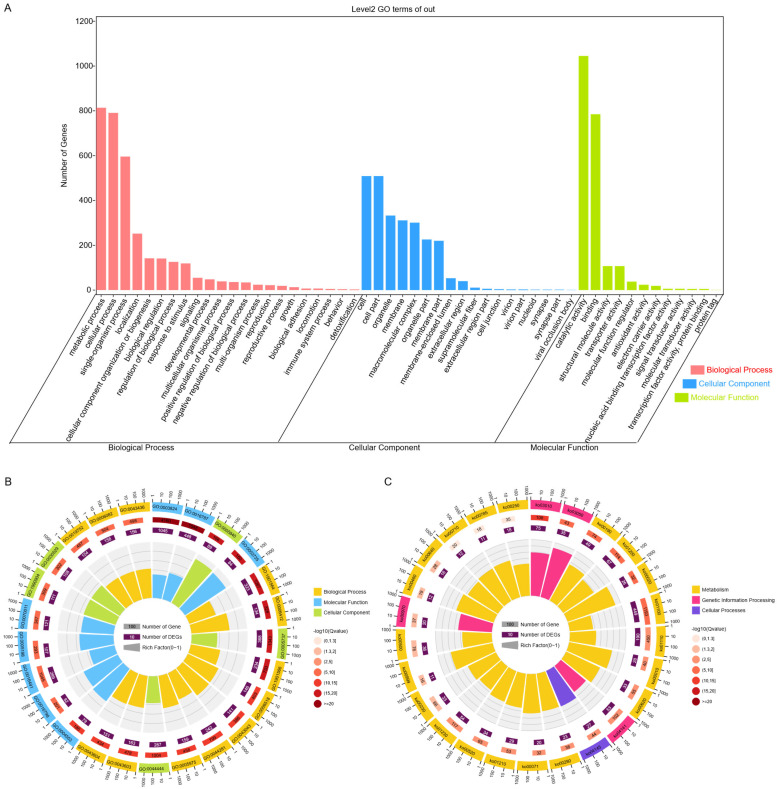
Proteomic profiling of PTR-EVs. (**A**) Level-2 GO term distribution based on protein counts. (**B**) GO functional classification of identified proteins. (**C**) KEGG pathway classification of identified proteins.

SDS–PAGE analysis of PTR-EV proteins showed clear and reproducible banding patterns with minimal background across three replicates (Figure S2). Consistently, the BCA assay measured an average protein concentration of 0.76 ± 0.18 mg/mL, supporting acceptable sample consistency for downstream proteomic analysis. As shown in Figure 2A, Level-2 GO annotation indicated that the PTR-EV proteome covered the three major GO domains: Biological Process (BP), Cellular Component (CC), and Molecular Function (MF). Within BP, metabolic process (GO:0008152; 814 proteins) and cellular process (GO:0009987; 791 proteins) were the most abundant categories, followed by single-organism process (GO:0044699; 596 proteins) and localization (GO:0051179; 252 proteins). GO enrichment analysis further highlighted translation (GO:0006412; *q* = 6.73 × 10^−18^), peptide biosynthetic process (GO:0043043; *q* = 3.57 × 10^−14^), and organonitrogen compound metabolic process (GO:1901564; *q* = 5.88 × 10^−18^) (Figure 2B).

Within CC, proteins were predominantly assigned to cell/cell part (GO:0005623/GO:0044464; 509 proteins each) and organelle (GO:0043226; 333 proteins). Enriched cellular locations included cytoplasm (GO:0005737; *q* = 1.24 × 10^−16^), ribosome (GO:0005840; *q* = 1.45 × 10^−20^), and ribonucleoprotein complex (GO:0030529; *q* = 9.11 × 10^−10^). Membrane-related assignments were also common, including membrane (GO:0016020; 311 proteins) and vesicle-associated membrane terms such as membrane-bounded vesicle (GO:0031988) and extracellular vesicle-related categories.

Within MF, catalytic activity (GO:0003824; 1045 proteins) and binding (GO:0005488; 785 proteins) were the dominant categories. Strong enrichment was observed for hydrolase activity (GO:0016787; *q* < 1 × 10^−20^), together with oxidoreductase activity (GO:0016491; *q* = 3.24 × 10^−10^), peptidase activity (GO:0008233; *q* = 2.08 × 10^−9^), structural constituent of ribosome (GO:0003735; *q* = 1.19 × 10^−18^), and unfolded protein binding (GO:0051082). Overall, the GO results indicated broad representation of proteins related to translation, proteostasis, membrane-associated localization, and catalytic functions.

#### 3.3.2. KEGG Annotation and EV-Associated Features of the PTR-EV Proteome

A total of 839 proteins were mapped to 96 KEGG pathways spanning metabolism, genetic information processing, cellular processes, and environmental information processing. The most highly represented pathways were metabolic pathways (ko01100; 449 proteins), biosynthesis of secondary metabolites (ko01110; 190 proteins), and carbon metabolism (ko01200; 67 proteins). As shown in Figure 2C and Figure S3, KEGG enrichment analysis (*q* < 0.05) identified ribosome (ko03010; 75 proteins; *q* = 1.06 × 10^−13^) and proteasome (ko03050; 35 proteins; *q* = 2.24 × 10^−9^) as the most significantly enriched pathways. Additional enriched pathways included oxidative phosphorylation (ko00190; 49 proteins), carbon metabolism (ko01200; 67 proteins), citrate cycle (ko00020; 26 proteins), glycolysis/gluconeogenesis (ko00010; 28 proteins), protein processing in the endoplasmic reticulum (ko04141; 53 proteins), and phagosome (ko04145; 27 proteins), all with *q* < 0.001.

Proteins associated with EV-related biogenesis or trafficking pathways were also detected, although the corresponding pathways did not reach enrichment significance. These included components assigned to soluble NSF attachment protein receptor (SNARE) interactions in vesicular transport (ko04130), autophagy–yeast (ko04138), endocytosis (ko04144), peroxisome (ko04146), and MAPK signaling–yeast (ko04011).

At the gene level, several proteins with reported relevance to EV associated processes in other systems were identified [38]. These included MAPK-related proteins such as a 14–3–3 protein (YWHAE; K06630; *GME3199_g*), an ERK-like MAPK (K04371; *GME10574_g*), and MAPK7 (K04464, *GME7057_g*), which aligns with stress sensing and downstream regulation. Proteins involved in central carbon and lipid metabolism were also detected, including hexokinases HK (K00844; *GME3755_g*, *GME8920_g*), 6-phosphofructokinase PFK (K00850; *GME2385_g*), and acetyl-CoA carboxylase ACACA (K11262; *GME12044_g*); and ER-associated folding and quality-control proteins, such as HSP90B (K09487; *GME3696_g*) and PDIA1 (K09580; *GME1349_g*), were detected, together with autophagy-linked proteins Vps15 (K08333; *GME2012_g*) and Atg8 (K08341; *GME6153_g*), supporting overlap between EV cargo selection and autophagy/ multivesicular body (MVB)-related routes [39]. Endosomal sorting complex required for transport (ESCRT)-associated components, including Vps32 (K12191; *GME6121_g*) and Vps4 (K12196; *GME6818_g*), were also present, supporting membrane invagination and vesicle scission. Vesicle fusion and trafficking were further supported by SNARE proteins VTI1 (K08493; *GME1554_g*) and SEC22 (K08517; *GME3639_g*), as well as Rab GTPases involved in secretory and endosomal pathways (RAB8A, Rab11A, Rab5B, Rab7A; *GME8416_g*, *GME5819_g*, *GME8733_g*, *GME8200_g*).

Proteins associated with cell wall-related carbohydrate metabolism were also prominent. Within amino sugar and nucleotide sugar metabolism (ko00520; 34 proteins), representative proteins included UDP-glucose pyrophosphorylase UGP2 (K00963; *GME11277_g*), multiple chitin synthases CHS1 (K00698; *GME3683_g*, *GME1843_g*, *GME11100_g*, *GME3387_g*, *GME748_g*, *GME2938_g*, *GME2813_g*), β-1,3-glucan synthase FKS1 (K00700; *GME3038_g*), and chitinase (K01183; *GME11412_g*).

Antioxidant and redox-related proteins included superoxide dismutases SOD2 (K04564; *GME10955_g*, *GME3756_g*, and *GME9084_g*), catalase CAT (K03781; *GME2693_g*), peroxiredoxin PRDX2/4 (K03386; *GME5191_g*), cytochrome c peroxidase (K00428; *GME7524_g*), glutathione S-transferase GST (K00799; *GME7757_g*), and manganese peroxidases (K20205; *GME10380_g*, *GME9085_g*, *GME9826_g*). Enzymes supporting redox cycling included glutathione reductase GSR (K00383; *GME5921_g*) and thioredoxin reductase trxB (K00384; *GME8494_g*), consistent with the glutathione metabolism (ko00480). Proteins linked to secondary metabolite biosynthesis were also detected, including phenylalanine ammonia-lyase PAL (K10775; *GME11328_g*) and enzymes of the terpenoid backbone pathway, such as acetyl-CoA C-acetyltransferase ACAT (K00626; *GME3453_g*), farnesyl diphosphate synthase FDPS (K00787; *GME5401_g*), and squalene synthase FDFT1 (K00801; *GME5396_g*). Together, these results indicate that PTR-EVs contain proteins associated with translation, proteostasis, trafficking, cell wall-related metabolism, and redox-related functions.

### 3.4. miRNA Analysis of PTR-EVs

Small RNA sequencing identified multiple miRNAs in PTR-EVs with predicted targets in the PTR genome (Table S3). However, the predicted targets were restricted to a relatively small gene set, including *GME317_g*, *GME2987_g*, *GME5969_g*, *GME7569_g* and *GME8540_g*, which were further analyzed by GO and KEGG annotation.

Level-2 GO classification assigned these targets mainly to metabolic process (GO:0008152; 4 genes), single-organism process (GO:0044699; 4 genes) and cellular process (GO:0009987; 3 genes), with additional assignments to biological regulation (GO:0065007; 2 genes) and cellular component organization or biogenesis (Figure S4). Within MF, catalytic activity (GO:0003824; 4 genes) and binding (GO:0005488; 3 genes) were the predominant categories, whereas CC annotations placed the targets in cell/cell part, organelles, membrane-related compartments, and macromolecular complexes.

Target-specific GO enrichment revealed functional differentiation among the predicted targets (Figure S5). *GME5969_g* was significantly enriched in glutamate synthase (NADH) activity (GO:0016040; *q* = 0.0198), together with oxidoreductase and cofactor-binding terms, supporting its role in nitrogen metabolism and redox balance. *GME8540_g* showed enrichment in receptor-binding terms, including dopamine receptor binding (GO:0050780; *q* = 0.0198) and G protein-coupled receptor binding (GO:0001664), and was associated with endoplasmic reticulum membrane-related localization. *GME317_g* was enriched in ubiquitin-conjugating enzyme activity (GO:0061631) and other ubiquitin pathway-related terms, whereas *GME2987_g* retained helicase-related annotations. *GME7569_g* was assigned to polygalacturonase activity (GO:0004650), consistent with polysaccharide degradation.

KEGG pathway mapping assigned four target to defined metabolic or regulatory pathways (Table S4). *GME5969_g* (K00264; NADH-dependent glutamate synthase) mapped to nitrogen metabolism (ko00910), alanine/aspartate/glutamate metabolism (ko00250), and amino acid biosynthesis (ko01230). *GME7569_g* (K01184; polygalacturonase) was linked to pentose and glucuronate interconversions (ko00040). *GME317_g* (K10583) mapped to ubiquitin-mediated proteolysis (ko04120). *GME2987_g* did not map to a significant KEGG pathway but retained functionally supported helicase-related GO annotations. Overall, the small RNA results provided a complementary molecular layer to the PTR-EV proteomic profile, although the number of predicted targets was limited and the miRNA evidence was interpreted as supplementary rather than mechanistic.

### 3.5. Factors Influencing PTR-EV Production

**Figure 3:**
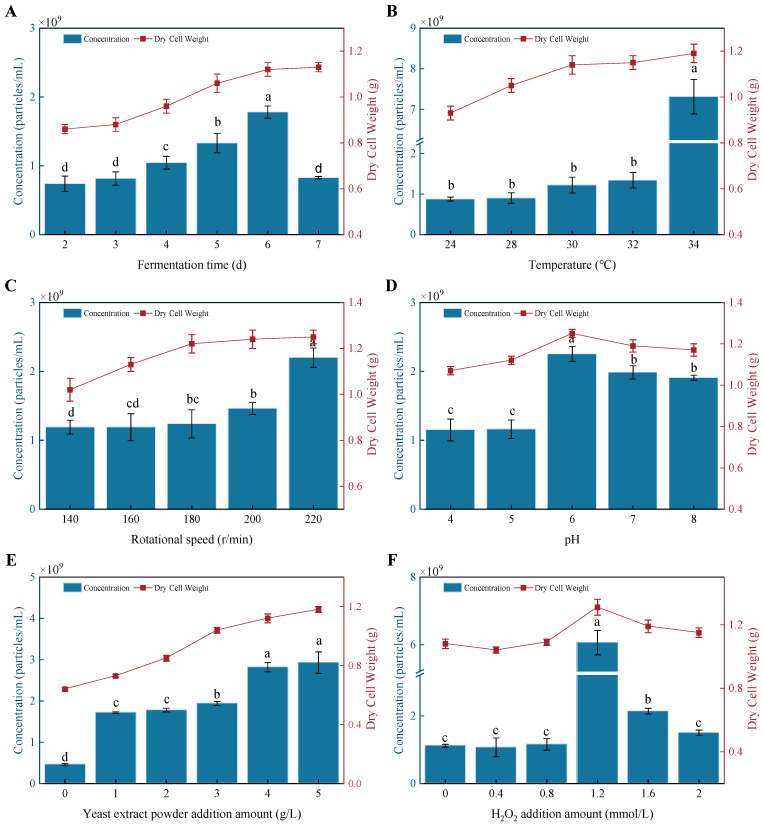
Effects of culture conditions on PTR-EV production. Changes in EV particle concentration and mycelial dry weight under different fermentation parameters, include (**A**) fermentation time, (**B**) temperature, (**C**) rotational speed, (**D**) pH, (**E**) yeast extract powder addition amount, and (**F**) H_2_O_2_ addition amount. Bars with different letters differ significantly (*p* < 0.05), whereas those with the same letter do not.

EV production was assessed by NTA particle concentration and mycelial dry weight. As shown in Figure 3A, EV concentration increased from day 2 to day 6 of fermentation and generally paralleled biomass accumulation. EV production was higher during the active growth phase and decreased at later stages as biomass approached a plateau. The maximum EV concentration was observed on day 6, reaching (1.78 ± 0.09) × 10^9^ particles/mL, and this time point was selected for subsequent experiments.

Temperature showed the strongest effect on EV yield (Figure 3B). Increasing the cultivation temperature to 34 °C led to a sharp rise in EV concentration, from 1.22 × 10^9^ to 7.31 × 10^9^ particles/mL, representing an approximately six-fold increase. One-way ANOVA confirmed a significant effect of temperature on EV production (*F*(4, 10) = 426.31, *p* = 4.02 × 10^−11^, *η*^2^ = 0.99).Tukey’s post-hoc test further showed that the yield at 34 °C was significantly higher than that at 30 °C (*p* = 1.74 × 10^−10^, *d* = 18.4). This marked increase in EV production at 34 °C is therefore consistent with a temperature-associated change in vesicle release.

Other culture parameters also affected EV production (Figure 3C–E). Increasing agitation to 220 r/min elevated EV concentration to 2.2 × 10^9^ particles/mL. EV concentration increased as initial pH rose from 4.0 to 6.0 but declined at higher pH values. Yeast extract supplementation enhanced EV production in a dose-dependent manner, reaching 2.93 × 10^9^ particles/mL at 5 g/L. Across these conditions, biomass trends generally paralleled EV concentration.

Oxidative stress altered EV generation (Figure 3F). H_2_O_2_ addition increased EV concentration in a dose-dependent manner up to 1.2 mmol/L, at which a maximum of 6.06 × 10^9^ particles/mL was observed, representing a 5.4-fold increase relative to the untreated control. At higher H_2_O_2_ levels, EV concentration declined. These data indicate that oxidative stimulation enhanced EV production only within a limited range. Taken together, these results identify temperature as the dominant factor within the tested ranges and under the present screening design.

### 3.6. RNA-Seq Analysis of Temperature Effects on PTR-EV Production

#### 3.6.1. GO Analysis

**Figure 4:**
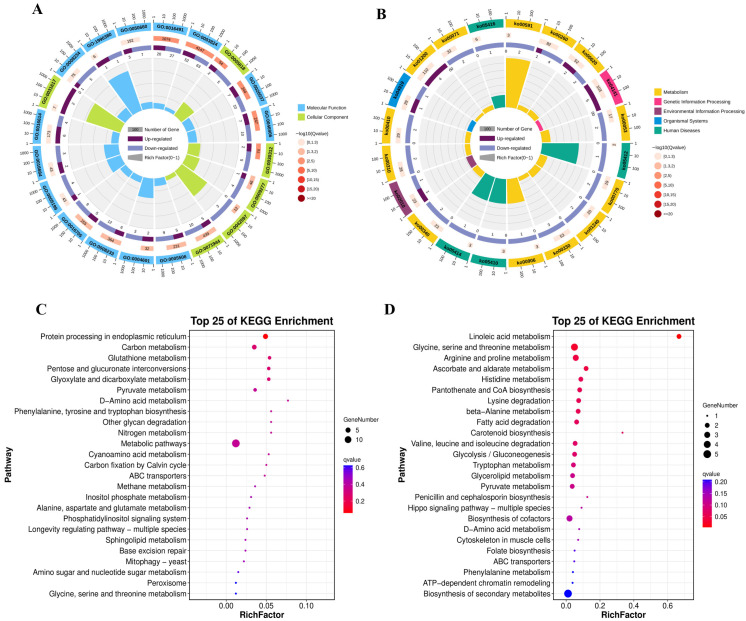
Transcriptomic response to elevated temperature in PTR. (**A**) GO classification of all significant DEGs. (**B**) KEGG pathway classification of DEGs. (**C**) Enriched KEGG pathways among upregulated DEGs. (**D**) Enriched KEGG pathways among downregulated DEGs.

Increasing the culture temperature from 30 °C to 34 °C resulted in 333 DEGs (*q* < 0.05; |log_2_FC| ≥ 1), including 148 up-regulated and 185 down-regulated genes (Figures S6 and S7). GO enrichment analysis highlighted two major functional groups relevant to temperature-responsive cellular remodeling: cell wall-related functions and redox-related functions (Figure 4A).

Down-regulated DEGs showed strong enrichment for cell wall-linked terms, including cell wall (GO:0005618), fungal-type cell wall (GO:0009277) and external encapsulating structure (GO:0030312), each contained 4–5 repressed genes, such as *GME1608_g*, *GME2725_g*, *GME3794_g*, and *GME6906_g* (Figure S8), which are linked to β-glucan/chitin organization. Redox-related GO terms, were also significantly enriched among the down-regulated genes, including oxidoreductase activity (GO:0016491), heme binding (GO:0020037) and tetrapyrrole binding (GO:0046906), included 10–26 down-regulated genes encoding putative oxidoreductases, such as *GME10336_g*, *GME10337_g*, and *GME4253_g*. In contrast, up-regulated DEGs showed fewer significant GO terms (Figure S9), yet the annotations included membrane transport and stress response, such as an ABC transporter–related annotation (*GME8425_g*) and stress-associated complex genes (*GME6875_g* and *GME691_g*).

#### 3.6.2. KEGG Analysis

KEGG enrichment revealed limited pathway enrichment among the up-regulated DEGs but strong pathway-level repression (Figure 4B). Among up-regulated DEGs, protein processing in endoplasmic reticulum (ko04141) was the only pathway near significance (5 genes; *q* = 0.058), including the membrane-bound site-2 protease S2P (*GME2708_g*; log_2_FC = 1.04) and four HSP20 family genes (*GME10364_g*, *GME10368_g*, *GME10370_g*, *GME6432_g*; log_2_FC 1.07–1.56) (Figure 4C).

Down-regulated DEGs showed significant enrichment in linoleic acid metabolism (ko00591; 2 genes; *q* = 0.00399), represented by 10R-lipoxygenases 10R-LOX (*GME10336_g*; log_2_FC = –1.38 and *GME10337_g*; log_2_FC = –1.63) (Figure 4D). Additional enriched pathways included glycine/serine/threonine metabolism (4 genes; *q* = 0.026), arginine/proline metabolism (3 genes; *q* = 0.043), and ascorbate/aldarate metabolism (2 genes; *q* = 0.043). Representative repressed genes in these pathways included DAAO (*GME1580_g*; log_2_FC = –1.86), 3-HAD family genes (*GME5391_g*, *GME2423_g*, *GME5405_g*; log_2_FC = –1.27 to –1.03), and ALDH(NAD^+^) isoforms (*GME6609_g*; log_2_FC = –1.72; *GME10646_g*, log_2_FC = –1.30).

#### 3.6.3. Integrative Analysis of Transcriptomic Remodeling and EV-Associated Protein Overlap

To integrate the RNA-seq dataset with EV proteomics and EV production phenotypes under elevated temperature, thermoresponsive DEGs were compared with proteins detected in PTR-EVs. Relative to 30 °C, cultivation at 34 °C markedly increased EV output, whereas biomass changed only slightly. Under the DEG threshold used above (FDR < 0.05 and |log_2_FC| ≥ 1), 333 thermoresponsive genes were identified, including 148 up-regulated and 185 down-regulated genes. Cross-comparison with the EV proteome identified 67 thermoresponsive genes whose protein products were also detected in PTR-EVs, and this overlap was greater than expected by chance (Fisher’s exact test, *p* = 8.89 × 10^−4^). Among these overlapping genes, 46 were down-regulated and 21 were up-regulated.

Based on transcriptomic annotation and overlap with the EV proteome, these overlapping molecules were organized into several interpretive functional modules (Table 2). Representative examples included a cell-envelope remodeling-related module containing wall- or membrane-associated factors such as *GME2725_g* and *GME10337_g*, a redox/lipid-related module including *GME10336_g* and other oxidoreductase-associated genes, and a proteostasis/stress-related module containing factors such as the HSP20 family-related protein *GME6432_g* and the stress-associated factor *GME6875_g*. Additional overlapping molecules were assigned to transport- or structure-related categories. Table 2 summarizes representative overlapping genes/proteins within these modules rather than the full list of 67 overlapping molecules.

#### 3.6.4. RT-qPCR Validation

To validate the RNA-Seq results, eight temperature-responsive DEGs were analyzed by RT-qPCR using a screening criterion of *q* < 0.05 and |log_2_FC| ≥ 1.0. The selected genes included four up-regulated targets (*GME691_g*, *GME829_g*, *GME8425_g*, and *GME10364_g*) and four down-regulated targets (*GME2725_g*, *GME4253_g*, *GME9069_g*, and *GME10337_g*). Primer sequence for these genes and the PTR-18S reference gene are listed in Table S1. As shown in Figure S10, the RT-qPCR results were in good agreement with the RNA-Seq data. For up-regulated genes, the log_2_-scale changes obtained by RT-qPCR were comparable to those from the transcriptomic analysis: *GME691_g* (0.93 vs. 1.11), *GME829_g* (2.99 vs. 3.89), *GME8425_g* (1.78 vs. 1.59), and *GME10364_g* (1.58 vs. 1.34). Similarly, the down-regulated genes showed consistent patterns between the two methods: *GME2725_g* (−1.04 vs. −1.30), GME4253_g (−1.75 vs. −1.68), *GME9069_g* (−4.14 vs. −5.69), and *GME10337_g* (−1.62 vs. −1.63). These results further support the reliability of the transcriptomic dataset.

### 3.7. Antioxidant Activity of PTR-EVs

**Figure 5:**
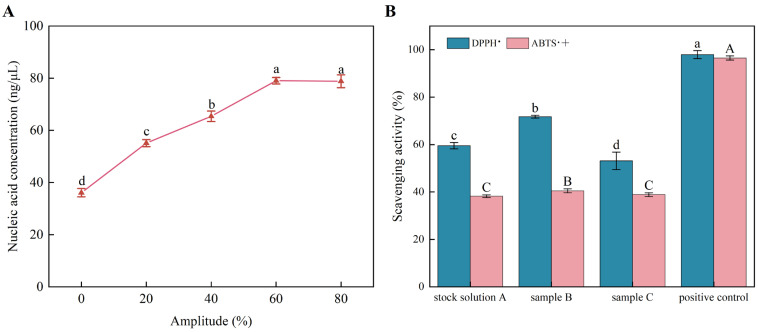
Effects of ultrasonic treatment on EV disruption and antioxidant activity. (**A**) Nucleic acid release from PTR-EVs at different ultrasonic power levels (total power 900 W). (**B**) Antioxidant activity of EVs under different treatments: PBS resuspension (Sample A), sonication (Sample B), and Triton X-100 treatment (Sample C). For bars of the same color, sharing the same lowercase (or uppercase) letter indicates no significant difference, and different letters indicate a significant difference (*p* < 0.05).

Ultrasonic power affected vesicle disruption as reflected by nucleic acid release (Figure 5A). Nucleic acid concentration increased with power and reached 79.0 ± 1.2 ng/μL at 60% power, with no further increase at 80% (78.8 ± 2.5 ng/μL). Based on this plateau, 60% power was selected for Sample B for subsequent assays. PTR-EVs prepared as intact vesicles (Sample A), ultrasonicated vesicles (Sample B), and Triton X-100–treated vesicles (Sample C) were evaluated for radical scavenging activity (Figure 5B). Sample B displayed the highest mean activity, with DPPH and ABTS scavenging rates of 71.73% ± 0.60% and 40.51% ± 0.81%, respectively. One-way ANOVA confirmed that DPPH radical scavenging differed significantly among the three treatment groups (*F*(2, 6) = 51.54, *p* = 1.68 × 10^−4^, *η*^2^ = 0.945). ABTS radical scavenging also differed significantly across groups (*F*(2, 6) = 7.80, *p* = 0.0214, *η*^2^ = 0.722). In both assays, Sample B showed the highest mean value among the tested groups.

### 3.8. Encapsulation Performance of PTR-EVs

As illustrated in Table 3, EE differed by cargo and loading method. Proanthocyanidins showed the highest mean EE under sonication (32.67% ± 1.3%), whereas curcumin showed the highest mean EE under co-incubation (49.83% ± 0.7%). One-way ANOVA confirmed that encapsulation efficiency of proanthocyanidins differed significantly among the three loading methods (*F*(2, 6) = 69.91, *p* = 6.97 × 10^−5^, *η*^2^ = 0.97). For curcumin, encapsulation efficiency also differed significantly across methods (*F*(2, 6) = 777.86, *p* = 5.67 × 10^−8^, *η*^2^ = 0.996). For both co-incubation and sonication, curcumin showed higher EE than proanthocyanidins.

**Figure 6:**
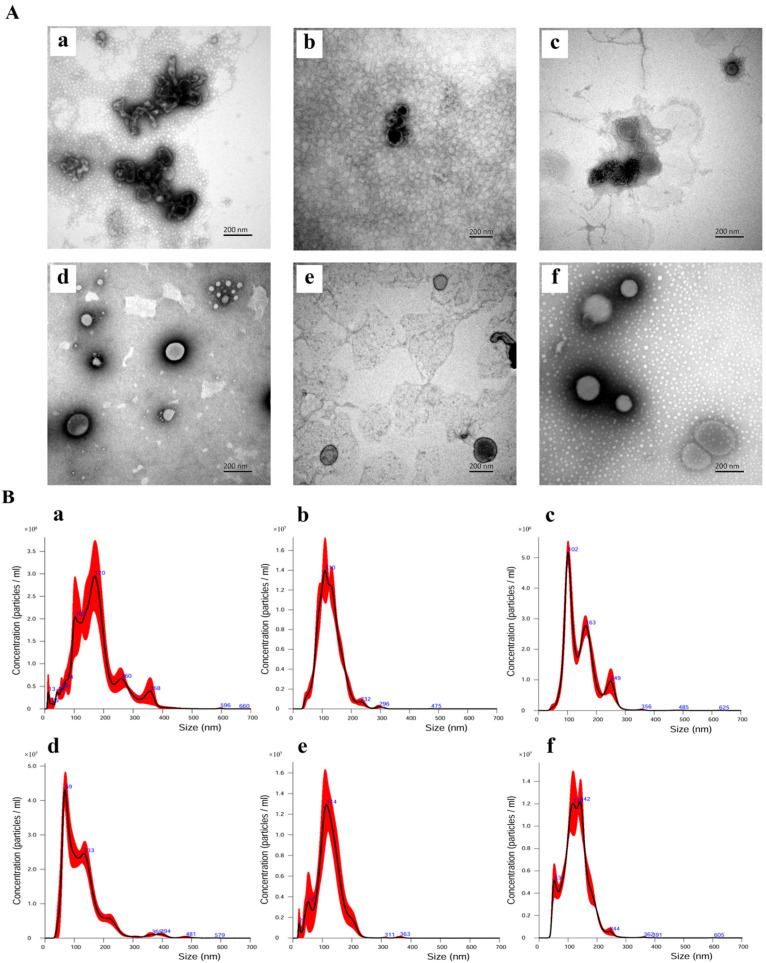
Effects of encapsulation methods on EV structure and size distribution. (**A**) Representative TEM images of EVs loaded with proanthocyanidins or curcumin using different methods. (**B**) Corresponding NTA size distribution profiles for the same samples shown in (**A**). Panels (**a**–**f**) in both (**A**,**B**) represent identical samples as follows: (**a**) proanthocyanidin–co-incubation; (**b**) proanthocyanidin–sonication; (**c**) proanthocyanidin–Triton X-100 treatment; (**d**) curcumin–co-incubation; (**e**) curcumin–sonication; (**f**) curcumin–Triton X-100 treatment. Scale bars = 200 nm. The red curve shows the measured particle size distribution, the black curve shows the fitted distribution, and the blue labels indicate the peak particle sizes (nm).

TEM images showed loading method-dependent differences in vesicle morphology (Figure 6A). For proanthocyanidin-loaded EVs, co-incubation was associated with vesicle shrinkage and crumpling in some fields, whereas sonication produced partially damaged vesicles (Figure 6A(b)). Triton X-100 treatment resulted in more extensive vesicle disruption and particle agglomeration. These observations were broadly consistent with the NTA results (Table 4). Proanthocyanidin-loaded EVs prepared by co-incubation or Triton X-100 treatment showed larger particle sizes (168.8 ± 8.2 nm and 146.2 ± 3.8 nm, respectively) together with lower particle concentrations. In contrast, sonication produced preparations with a clearer single peak and a more concentrated size distribution (Figure 6B(b)).

For curcumin-loaded EVs, TEM images showed more evident particle-associated curcumin under co-incubation and Triton X-100 treatment (Figure 6A(d–f)). NTA analysis further showed that curcumin-loaded EVs prepared by co-incubation or Triton X-100 treatment had larger particle sizes than those prepared by sonication (Table 4).

### 3.9. Stability of Encapsulated PTR-EVs

**Figure 7:**
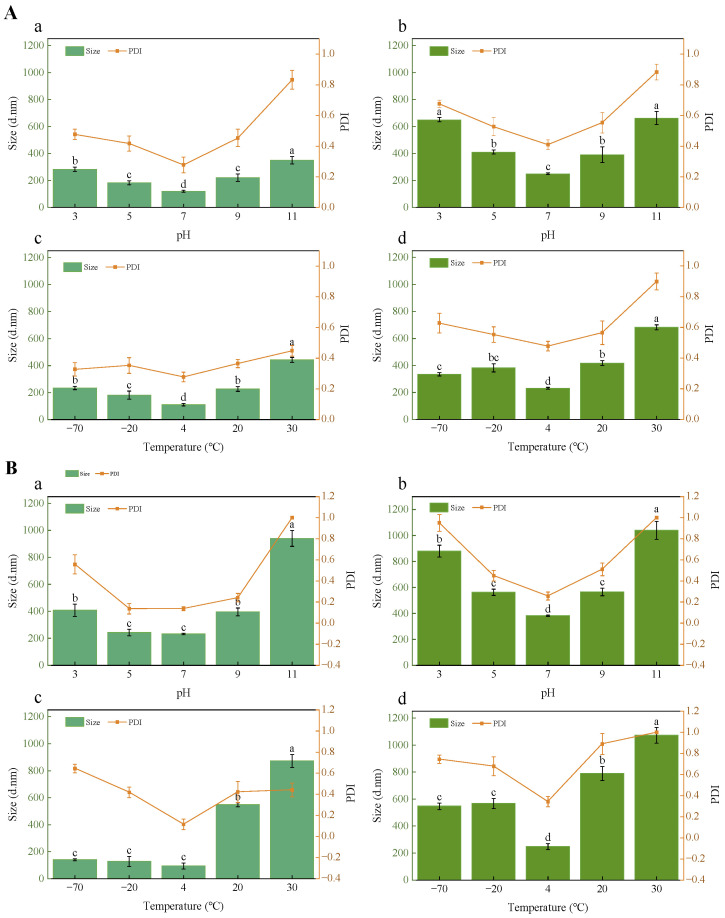
Particle size and PDI of loaded PTR-EVs under different pH and temperature conditions. Proanthocyanidin-loaded EVs (**A**) and curcumin-loaded EVs (**B**) were evaluated after 24 h (**a**,**c**) and 72 h (**b**,**d**) under the indicated pH and temperature conditions. Different letter labels indicate the results of the multiple-comparison test within each individual panel. Bars marked with different letters within the same panel are significantly different (*p* < 0.05), whereas bars sharing the same letter within the same panel are not significantly different.

**Figure 8:**
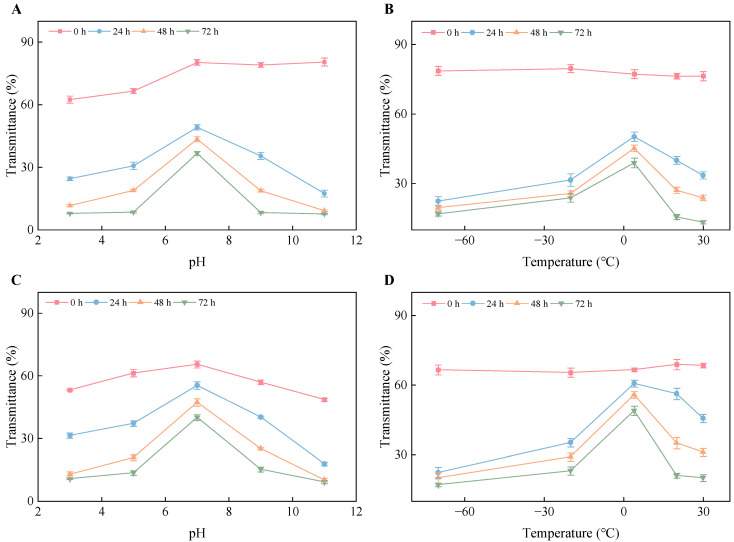
Time-dependent transmittance changes of loaded PTR-EVs under different pH and temperature conditions. Transmittance of proanthocyanidin-loaded EVs (**A**,**B**) and curcumin-loaded EVs (**C**,**D**) was monitored over 72 h under the indicated pH and temperature conditions.

Physical stability was further monitored over 72 h by transmittance, particle size, and PDI under different pH and temperature conditions (Figure 7 and Figure 8). For both proanthocyanidin-loaded EVs and curcumin-loaded EVs, transmittance decreased over time under all tested conditions, but the decrease was smallest at pH 7 and at 4 °C. In contrast, acidic, alkaline, freezing, and elevated-temperature conditions caused more pronounced reductions in transmittance. Particle size and PDI measurements were in agreement with the transmittance results. At both 24 h and 72 h, lower particle size and PDI were observed at pH 7, whereas pH 11 showed the largest increases in both parameters. These differences became more evident at 72 h, when strongly alkaline conditions produced substantial particle enlargement and a marked increase in PDI. Under temperature treatment, 4 °C maintained comparatively smaller particle size and lower PDI, whereas 30 °C was associated with greater particle growth and broader size distribution. Freezing conditions (–70 and –20 °C) also affected stability, particularly in transmittance and PDI. These results show clear time- and condition-dependent changes in the short-term physical stability of the loaded EV systems under the tested model conditions.

## 4. Discussion

### 4.1. Isolation Strategy, EV Identity, and Molecular Features of PTR-Derived Vesicle Preparations

PTR-EVs were isolated from both fermentation broth and enzymatic digests, consistent with the reported localization of fungal EVs in the periplasmic space between the cell wall and plasma membrane [40]. Repeated experiments confirmed that lywallzyme effectively degraded the cell wall of higher fungi while intact protoplasts were preserved under osmotic protection, indicating that vesicles released during enzymatic treatment could still be operationally classified as extracellular vesicles [28]. Among the tested methods, differential centrifugation produced better size consistency than ultrafiltration, and fermentation broth yielded more homogeneous particle populations than enzymatic digests, in which broader size distributions likely reflected residual enzymes or incompletely removed wall-derived material. To reduce these sources of heterogeneity, subsequent analyses were conducted using vesicle preparations isolated from the fermentation broth. The observed size range of PTR-EVs (50–600 nm) was also consistent with previous fungal EV reports, including 30–1000 nm in *C. albicans* [41], 100–600 nm in *Aspergillus fumigatus* [42], and an average size of 315.21 ± 166.88 nm in *S. cerevisiae* [43].

At the same time, the present protocol relied mainly on differential centrifugation and did not include density gradient ultracentrifugation (dUC) as a higher-rigor purification step or benchmark [44]. Thus, although the resulting preparations were suitable for comparative compositional and preliminary functional characterization, co-isolated non-vesicular components, such as protein aggregates, membrane fragments, or cell wall-derived particles, cannot be fully excluded. The molecular and functional findings should therefore be interpreted with appropriate caution, particularly when assigning specific features exclusively to EVs themselves. Future studies incorporating dUC together with marker validation and more stringent exclusion of non-vesicular particles will be necessary to strengthen purity assessment and confirm the specificity of the reported EV-associated features.

A further challenge in higher-fungal EV research is the lack of broadly accepted markers. In animal EVs, markers such as CD9, CD63, CD81, TSG101, and Flotillin 1 are widely used [44]. Bacterial outer membrane vesicles (OMVs) can be identified through surface lipopolysaccharides (LPS) [45], whereas plant EV studies have proposed marker families, including fasciclin-like arabinogalactan proteins (FLAs), patellins, and germin-like proteins [46]. Within fungi, candidate markers have been reported in species such as *S. cerevisiae* and *C. albicans*, and Sur7 has recently been confirmed as a surface marker for EVs from *Fusarium graminearum* [47]. By contrast, widely accepted markers for EVs from higher fungi remain lacking. In the present study, KEGG orthology-guided analysis of the PTR-EV proteome identified several proteins homologous to EV-associated markers or EV-biogenesis-related components reported in other systems, including ESCRT-related proteins (CHMP2, VPS4), endosomal trafficking regulators (Rab7, VPS35), and heat shock proteins (HSP40, HSP70, HSP90), mainly assigned to KEGG pathways 04144 (Endocytosis) and 04141 (Protein processing in ER) (Table S5). These findings support the presence of evolutionarily conserved EV-related machinery in PTR, but they do not constitute experimental validation of PTR-specific EV markers.

Proteomic profiling further showed that PTR-EVs contained proteins associated with translation, proteostasis, central metabolism, membrane trafficking, cell wall-related metabolism, and redox-associated functions. Enrichment of ribosomal proteins, protein quality-control elements, and vesicle-associated membrane terms in the GO annotations was consistent with an endomembrane-linked origin [48,49]. KEGG analysis also revealed strong representation of oxidative phosphorylation, carbon metabolism, the TCA cycle, glycolysis/gluconeogenesis, ER protein processing, and phagosome pathways. Although several trafficking-related pathways did not reach formal enrichment significance, the detection of ESCRT components, Rab GTPases, SNARE proteins, and autophagy-related factors was more consistent with vesicle-associated trafficking than with a purely nonspecific leakage model [50]. PTR-EVs also contained both cell wall synthases and hydrolases, supporting a possible vesicle-associated role in cell wall remodeling [51], together with antioxidant enzymes linked to cellular stress management [52]. Small RNA analysis provided a complementary molecular layer, with predicted target functions broadly aligned with nitrogen metabolism, ubiquitin-mediated proteolysis, redox-related processes, and polysaccharide degradation. However, because the number of predicted targets was limited and no direct target validation was performed, the small RNA evidence should be regarded as supplementary and correlative rather than mechanistic.

### 4.2. Environmental Regulation of PTR-EV Production and Temperature-Associated Remodeling

**Figure 9:**
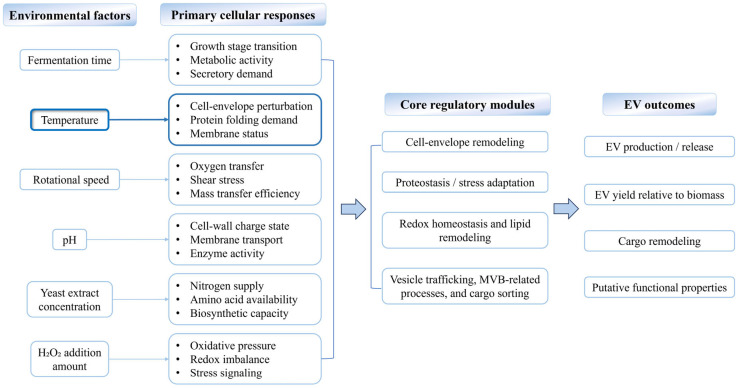
Conceptual framework linking environmental factors to PTR-EV production, cargo remodeling, and putative functional properties. Multiple environmental cues, including fermentation time, temperature, rotational speed, pH, yeast extract concentration, and H_2_O_2_ addition, may trigger partially overlapping cellular responses. These responses converge on broader regulatory modules related to cell-envelope remodeling, proteostasis/stress adaptation, redox and lipid metabolic reprogramming, and vesicle trafficking/cargo sorting, which may in turn be associated with changes in EV biogenesis/release, EV yield per biomass, cargo remodeling, and putative functional properties. Temperature is highlighted because it showed the strongest effect on EV production in the present study.

Beyond molecular composition, PTR-EV production was responsive to multiple environmental factors, including fermentation time, temperature, rotational speed, pH, yeast extract concentration, and H_2_O_2_ supplementation, suggesting modulation through partially overlapping cellular responses rather than a single route. Among these variables, temperature exerted the strongest effect in the present study. EV concentration increased during active biomass accumulation and declined as growth approached a plateau, suggesting a close association between vesicle release, growth stage, and metabolic activity, consistent with observations in *A. fumigatus* and *Trichoderma harzianum* [28,53]. Likewise, cultivation at 34 °C markedly increased the yield of vesicle-like particles, whereas excessive H_2_O_2_ reduced particle output, suggesting that vesicle release was favored within a permissive stress range rather than under unrestricted stress intensity [54,55,56]. More broadly, these effects can be interpreted within a working framework in which growth state, membrane/envelope status, nutrient supply, oxygen transfer, pH-dependent transport properties, and oxidative pressure converge on core modules related to cell-envelope remodeling, proteostasis/stress adaptation, redox and lipid metabolic reprogramming, and vesicle trafficking/cargo sorting (Figure 9).

At the same time, the observed increase in vesicle-like particles under elevated temperature should be interpreted cautiously. The present data do not allow a clear distinction between actively regulated vesicle biogenesis and passive release associated with altered membrane stability or envelope perturbation under heat stress. Thus, the temperature effect is better regarded as a biologically plausible working hypothesis rather than a definitive mechanistic conclusion. In this context, elevated temperature may promote regulated vesicle production, passive membrane shedding, or both.

Transcriptomic analysis under elevated temperature revealed predominant repression of genes related to cell wall organization and redox buffering, together with limited induction of stress-response and membrane-associated functions. Repression of β-glucan- and chitin-associated genes may reflect reduced cell wall reinforcement and therefore lower physical constraints on vesicle passage. At the same time, repression of oxidoreductase- and lipid-related pathways implies altered redox balance and membrane status [57], whereas induction of ER proteostasis-related genes, including S2P and members of the HSP20 family, is consistent with increased folding and quality-control demand under thermal stress [58]. These changes support the view that temperature altered the cellular context in which vesicle release occurred, but they do not by themselves establish the mechanism of vesicle generation.

Cross-analysis of RNA-seq and EV proteomics further suggested that the thermal transcriptional response was linked to PTR-EV cargo composition rather than remaining confined to intracellular gene expression changes. Proteins encoded by genes repressed at 34 °C were also detected in PTR-EVs (Table S6), a pattern compatible with non-random cargo partitioning, although not direct proof of cargo-selection mechanisms [59]. Together with the functional modules identified above, these data suggest that vesicle release under elevated temperature was accompanied by broader remodeling of cell-envelope properties, metabolic state, and proteostasis capacity [60]. However, these associations remain correlative, and further work will be needed to determine whether temperature directly regulates EV biogenesis pathways in PTR.

### 4.3. Preliminary Functional Evaluation of PTR-EVs as Edible Fungal Nanocarrier Candidates

Against this molecular and regulatory background, the DPPH and ABTS assays used here evaluate radical-scavenging capacity in chemical systems only and do not establish antioxidant efficacy in biological systems or in vivo. Within this limited framework, the measured antioxidant activity of PTR-EVs was strongly influenced by vesicle integrity and treatment method. Sonication produced the highest radical-scavenging activity, suggesting that partial vesicle disruption increased access to intravesicular antioxidant components. By contrast, Triton X-100 treatment did not yield comparable activity, which may reflect cargo loss, protein denaturation, or assay interference. These results should therefore be regarded as a preliminary physicochemical indication of potential antioxidant properties rather than as direct evidence of biological antioxidant function.

EVs from different biological sources differ substantially in safety, functionality, and production feasibility. Plant- and mammalian-derived EVs, particularly plant nanovesicles and milk-derived EVs, have been widely explored as carriers for drug delivery and oral delivery of bioactive compounds, and food-derived vesicles are increasingly discussed in the context of functional foods and precision nutrition [61,62,63]. In agriculture, EV-mediated cross-kingdom RNA transport and plant–microbe communication have also emerged as promising application-oriented directions for crop protection [64,65,66]. By comparison, fungal EVs remain much less studied, and recent reviews continue to highlight major gaps in isolation methods, marker definition, cargo interpretation, and translational application [67,68,69]. Recent reports on vesicles from edible mushrooms, including *Pleurotus eryngii* and shiitake, nevertheless indicate growing interest in fungal EVs as bioactive nanocarriers, although the field remains less mature than plant- or milk-derived EV research [70,71].

Within this context, PTR-EVs may offer several practical advantages. Because they are derived from an edible, GRAS-classified fungal system, they can be produced by animal-free fermentation with relatively controllable culture conditions and reduced biosafety concerns. In addition, unlike many plant EV systems whose antioxidant properties may depend on variable polyphenol content [72,73], PTR-EVs may contain endogenous antioxidant enzymes and redox-related proteins, potentially providing a distinct functional basis. Even so, this possibility remains preliminary in the absence of cell-based validation and more rigorous purification controls.

Cargo loading depended strongly on both cargo properties and loading strategy. Hydrophobic curcumin showed higher encapsulation efficiency under co-incubation, whereas hydrophilic proanthocyanidins loaded more efficiently under sonication. TEM and NTA analyses further showed that co-incubation and Triton X-100 treatment were often associated with vesicle deformation, aggregation, or broader size distributions, whereas sonication generally produced narrower distributions but partially compromised vesicle integrity. Thus, loading efficiency, structural preservation, and storage stability must be balanced rather than optimized independently.

Compared with many reported food-grade delivery systems, the encapsulation performance of PTR-EVs remained modest. The encapsulation efficiency for proanthocyanidins (32.67% ± 1.3%) was lower than values reported for anthocyanin-loaded liposomes (52.2%, 71%, and 81.28%) [74,75,76] and chitosan–pectin anthocyanin nanogels (66.7%) [75]. For curcumin, the highest encapsulation efficiency of PTR-EVs (49.83% ± 0.7%) was also lower than values commonly reported for liposomal systems (about 63–78%) or some polysaccharide-based nanogels and related biopolymer carriers (about 89–94%) [77]. Direct comparison across systems should nevertheless be interpreted cautiously, because carrier composition, cargo chemistry, loading protocols, and quantification methods differ among studies. PTR-EVs also showed only limited short-term stability under food-relevant pH and temperature conditions, with better performance at pH 7 and 4 °C but reduced stability under alkaline or elevated-temperature conditions. These findings indicate that PTR-EVs, in their current form, do not yet match the performance of established food delivery systems in terms of encapsulation efficiency and environmental stability [78]. Their potential value therefore lies less in immediate application readiness than in supporting the feasibility of edible fungal vesicle preparations as a starting platform that merit further optimization for food-related use.

### 4.4. Study Limitations and Outlook

Several limitations should be acknowledged. Vesicle purity cannot be considered definitive because the current purification strategy did not fully exclude co-isolated non-vesicular material. Thus, some molecular and functional features detected here may reflect mixed vesicle-enriched preparations rather than exclusively EV-specific components. The multi-omics results provide integrative and correlative support for EV-associated remodeling, but do not establish direct mechanisms of EV biogenesis, cargo sorting, or stress adaptation. In particular, the present study does not distinguish between active, regulated vesicle biogenesis and passive release associated with membrane perturbation under elevated temperature. The environmental-factor analysis was based on a one-factor-at-a-time screening design, which identified dominant variables but did not resolve interactions among factors. Functional evaluation also remained preliminary: antioxidant assays were limited to chemical assays, stability analysis reflected only short-term physicochemical behavior under model conditions, and the current controls did not fully separate EV-specific effects from possible contributions of co-isolated material. In addition, the study supports only a condition-linked association between temperature-responsive vesicle production and transcriptomic remodeling. Future work should prioritize higher-rigor purification, marker validation, matched multi-omic sampling, more rigorous functional controls, and cell-based or application-relevant validation systems.

## 5. Conclusions

This study shows that differential centrifugation can recover PTR-derived vesicle preparations from *P. tuber-regium* fermentation broth with relatively uniform particle characteristics and appreciable yield under the tested conditions. Multi-omics analyses indicated that these preparations contained diverse molecular components associated with protein turnover, membrane trafficking, cell-envelope functions, redox-related processes, and regulatory small RNAs. Vesicle production was responsive to multiple culture conditions, with temperature showing the strongest association in the present study. Integrative analysis further suggested that elevated temperature was associated with coordinated changes in cell-envelope remodeling, redox/lipid metabolism, proteostasis-related pathways, and vesicle-associated molecular composition, although the present data do not distinguish regulated vesicle biogenesis from passive release linked to membrane perturbation. Functionally, the vesicle preparations showed radical-scavenging activity in chemical assays, which increased after ultrasonic disruption, and were able to associate with both hydrophilic and hydrophobic model compounds. Short-term stability analyses identified pH 7 and 4 °C as relatively favorable conditions under the tested model conditions. Overall, these findings support PTR-derived vesicle preparations as a useful edible fungal model for investigating environmentally responsive vesicle biology as a preliminary platform for evaluating food-related nanoscale delivery potential. Further work will be needed to improve purification rigor, validate candidate EV-associated markers, clarify cargo specificity and functional mechanisms, and assess longer-term performance under application-relevant conditions.

## Figures and Tables

**Table 1:** DLS and NTA characterization of PTR-EVs isolated by different methods.

Sample Origin	Separation Method	DLS Particle Size (nm)	NTA Mean ^a^ (nm)	NTA Mode ^b^ (nm)	NTA Particle Concentration (×10^8^ Particles/mL)
Fermentation broth	Differential centrifugation	196.4 ± 19.3	125.0 ± 0.7	115.3 ± 6.3	12.3 ± 0.2
	Ultrafiltration	371.2 ± 99.0	168.7 ± 5.5	129.6 ± 5.5	5.2 ± 0.4
Enzymatic digestion	Differential centrifugation	247.8 ± 3.8	142.6 ±- 2.6	138.6 ± 9.5	7.2 ± 0.7
	Ultrafiltration	260.5 ± 18.3	145.0 ± 9.8	139.8 ± 1.2	6.3 ± 0.7

^a^ NTA mean: the arithmetic mean particle size, reflecting the overall size level of the sample. ^b^ NTA mode: the peak value in the size distribution, indicating the most common particle size. The proximity between mean and mode values indicates size distribution uniformity—closer values suggest a more concentrated and homogeneous sample.

**Table 2:** Integrated functional modules derived from overlap between thermoresponsive DEGs and PTR-EV proteins under elevated temperature.

Functional Module	Key Transcriptomic Evidence	Representative Overlapping Molecules	Potential Relevance to EV-Associated Remodeling
Cell-envelope remodeling	GO: cell wall; fungal-type cell wall; external encapsulating structure; mostly down-regulated	*GME2725_g*, *GME10337_g*	May be associated with reduced physical constraints on vesicle passage
Redox/lipid metabolic reprogramming	GO: oxidoreductase activity, heme binding; KEGG: linoleic acid metabolism; mostly down-regulated	*GME10336_g*, *GME10646_g*, *GME9500_g*	May be associated with changes in membrane/redox state relevant to EV production and cargo remodeling
Proteostasis/stress adaptation	KEGG: protein processing in ER; stress-related genes; mainly up-regulated	*GME6432_g*, *GME6875_g*, *GME10681_g*	May be associated with increased folding and quality-control demand during cargo processing
Transport/structural response	Transporter or structure-related annotations; mixed regulation	*GME7134_g*, *GME7852_g*	May be associated with trafficking-related or structural responses relevant to EV release
Cytoskeleton/structural dynamics	Functional annotation from overlap genes/proteins; down-regulated	*GME7852_g*	May reflect structural changes accompanying vesicle release

**Table 3:** Encapsulation efficiency of bioactive compounds in PTR-EVs.

Encapsulated Substance	Co-Incubation Group	Ultrasound Group	Triton X-100 Group
Proanthocyanidins	20.01% ± 0.6% b	32.67% ± 1.3% a	18.48% ± 2.4% b
Curcumin	49.83% ± 0.7% a	46.01% ± 0.5% b	17.42% ± 1.7% c

Different lowercase letters (a, b, c) indicate significant differences between groups (*p* < 0.05).

**Table 4:** NTA characterization of EVs prepared by different encapsulation methods.

Embedded Substances—Handling Methods	Particle Size (nm)	Particle Concentration (×10^8^ Particles/mL)
Proanthocyanidins—Co-incubation	168.8 ± 8.2	3.68 ± 0.82
Proanthocyanidins—Ultrasonication	125.6 ± 1.6	12.00 ± 0.34
Proanthocyanidins—Triton X-100	146.2 ± 3.8	3.78 ± 0.20
Curcumin—Co-incubation	125.0 ± 4.5	36.9 ± 3.62
Curcumin—Ultrasonication	119.9 ± 8.3	10.07 ± 0.28
Curcumin—Triton X-100	127.3 ± 17.0	11.90 ± 0.10

## Data Availability

The original contributions presented in this study are included in the article/Supplementary Materials. Further inquiries can be directed to the corresponding author.

## Supplementary Materials

The following supporting information can be downloaded at: https://www.mdpi.com/article/10.3390/foods15081439/s1, Table S1: primer sequences used for RT-qPCR validation; Table S2: EV particle concentrations in edible mushroom fermentation broths; Table S3: predicted target genes of PTR-EV–associated miRNAs; Table S4: KEGG pathway annotation of predicted miRNA target genes; Table S5: Candidate EV-associated biomarkers identified in PTR-EVs based on KEGG ortholo-gy-guided proteomic analysis; Table S6: Integrated analysis of PTR EVs proteome and temperature-responsive transcriptome; Figure S1: DLS profiles of EVs from different edible mushrooms; Figure S2: SDS-PAGE analysis of PTR-EV proteins; Figure S3: KEGG pathway enrichment analysis of proteins identified in PTR-EVs; Figure S4: Level-2 GO classification of predicted miRNA target genes; Figure S5: GO enrichment analysis of predicted miRNA target genes; Figure S6: Level-2 GO term distribution of temperature-responsive differentially expressed genes in PTR; Figure S7: Volcano plot of DEGs identified by RNA-Seq analysis; Figures S8: Top 20 enriched GO terms among downregulated DEGs; Figure S9: Top 20 GO enriched terms among upregulated DEGs; Figure S10: RT-qPCR validation of selected temperature-responsive DEGs.

## Abbreviations

The following abbreviations are used in this manuscript:

BCA	Bicinchoninic acid
BP	Biological process
CC	Cellular component
DEGs	Differentially expressed genes
DLS	Dynamic light scattering
EE	Encapsulation efficiency
ESCRT	Endosomal sorting complex required for transport
EVs	Extracellular vesicles
MF	Molecular function
MVB	Multivesicular body
NTA	Nanoparticle tracking analysis
PBS	Phosphate-buffered saline
PDI	Polymer dispersity index
PTR	*Pleurotus tuber-regium*
SNARE	Soluble NSF attachment protein receptor
TEM	Transmission electron microscopy
