# Supplementary material for: Environmental Regulation, Molecular Profiling, and Preliminary Functional Evaluation of Extracellular Vesicles from Pleurotus tuber-regium
Source: Foods. 2026 Apr 21;15(8):1439. doi: 10.3390/foods15081439 (PMC13115048; doi:10.3390/foods15081439)
Supplement: Supplementary file 1 [file foods-15-01439-s001.zip › Clean-R1-foods-4231308-supplementary material-20260418.pdf]

foods

MDPI

Supplementary material

Environmental Regulation, Molecular Profiling, and Preliminary Functional Evaluation of Extracellular Vesicles from *Pleurotus tuber-regium*

Wen Li [1,2], Junyi Fang [1,2], Xiaoyan Zhang [1,2], Mengmeng Xu [1,2], Peter C.K. Cheung [3], Guiyang Shi [1,2], Lei Chen [1,2,*] and Zhongyang Ding [1,2,]

[1] School of Biotechnology and Key Laboratory of Carbohydrate Chemistry and Biotechnology of Ministry of Education, Jiangnan University, Wuxi 214122, China
[2] National Engineering Research Center of Cereal Fermentation and Food Biomanufacturing, Jiangnan University, Wuxi 214122, China
[3] School of Life Sciences, The Chinese University of Hong Kong, Shatin, New Territories, Hong Kong, China
* Correspondence: leichen@jiangnan.edu.cn or superchen7@gmail.com; Tel.: +86-152-5168-5286

1. Supplementary Methods

1.1 EV proteomics workflows

1.1.1 Protein Extraction and Quantification

All EV protein samples were dried by rotary evaporation at 0°C, then dissolved in 70 μL RIPA lysis buffer. The samples were sonicated in an ice-water bath using a cell disruptor for 20 minutes, followed by centrifugation at 12,000 rpm and 4°C for 10 minutes. The supernatant was transferred to a new EP tube.

Protein quantification was performed using the BCA method. BCA protein standards of known concentrations were diluted with ultrapure water to 0.025, 0.05, 0.1, 0.2, 0.3, 0.4, and 0.5 mg/mL to generate a standard curve. BCA working solution (A:B = 50:1) was added to a 96-well plate at 200 μL per well, followed by 20 μL of sample (diluted accordingly) or standard protein. The plate was incubated at 37°C with shaking for 30 minutes, and absorbance was measured at 562 nm. Protein concentrations were calculated based on the standard curve.

1.1.2 Reduction, Alkylation, and Protein Digestion

A 10 μg protein aliquot (40 μL) was mixed with 10 μL of reduction/alkylation reagent (500 mM NH_4HCO_3, 25 mM TCEP, 50 mM CAA, 0.5% SDC) at a volume ratio of 4:1 (protein:reagent). The mixture was thoroughly mixed and incubated in a thermomixer at 95°C and 1,200 rpm for 10 minutes.

After the sample returned to room temperature, 2 μL of SP3 magnetic beads was added at a protein-to-bead ratio of 1:10 (w/w), followed by shaking at room temperature and 1,200 rpm for 2 minutes. Subsequently, 130 μL of anhydrous acetonitrile was added, and the sample was shaken at room temperature and 1,200 rpm for 1 minute, then allowed to stand at room temperature for 10 minutes. This step was repeated once. The sample was placed on a magnetic stand for 2 minutes to allow bead adsorption, and the supernatant was removed. The beads were washed sequentially with 180 μL of acetonitrile and 180 μL of 80% ethanol, allowing 20 seconds of standing each time before removing the liquid.

After the organic solvents had evaporated, 20 μL of 100 mM ammonium bicarbonate (pH 8.0) was added to the sample. The sample was shaken at room temperature and 1,200

rpm for 1 minute, followed by sonication for 20 seconds to ensure thorough mixing. Trypsin was added at an enzyme-to-protein ratio of 1:50 (w/w), and the sample was shaken at room temperature and 1,200 rpm for 1 minute, then incubated in a thermomixer at 37°C and 1,200 rpm with shaking for 2 hours. After digestion, the sample was briefly centrifuged, placed on a magnetic stand for 2 minutes, and the supernatant was collected. Peptides were eluted by adding 0.1% formic acid, followed by sonication at room temperature for 1 minute, incubation at room temperature and 1,300 rpm for 5 minutes, brief centrifugation, and another 2 minutes on a magnetic stand. The supernatant was collected for peptide concentration determination prior to mass spectrometry analysis.

1.1.3 NanoLC-MS/MS Analysis

Approximately 500 ng of peptides from each sample were separated using a nano-UPLC system (Vanquish Neo, Thermo Scientific) coupled to an Astral mass spectrometer (Thermo Scientific) equipped with a nano-electrospray ion source. Peptide separation was performed on a reversed-phase column (EASY-Spray™, 150 μm × 15 cm; Thermo Scientific, USA). Mobile phase A consisted of ultrapure water containing 0.1% formic acid, and mobile phase B consisted of 80% acetonitrile containing 0.1% formic acid. After equilibrating the column with 96% mobile phase A, samples were directly loaded onto the column by an autosampler for gradient separation.

Mass spectrometry analysis was performed in data-independent acquisition mode with positive ion detection. MS1 scans were acquired over an m/z range of 380–980 at a resolution of 240 K. MS2 scans were acquired over an m/z range of 150–2000, with an absolute AGC target of 5×10^4, a maximum injection time of 3 ms, an isolation window of 2 m/z, and higher-energy collisional dissociation at a normalized collision energy of 25%.

1.1.4 Database Search

Raw MS files were processed using Spectronaut software (version 19.0.240604.62635; Biognosys AG) for database searching. The protein sequence database was the UniProt database (uniprotkb_taxonomy_id_716892_2025_10_20.fasta). Search parameters were set as follows: trypsin was used as the enzyme, allowing up to 2 missed cleavages; carbamidomethylation of cysteine (+57.021 Da) was set as a fixed modification; acetylation of protein N-termini (+42.011 Da) and oxidation of methionine (+15.995 Da) were set as variable modifications; precursor mass tolerance was 20 ppm, and fragment mass tolerance was 20 ppm. The false discovery rate for both peptides and peptide-spectrum matches was controlled at ≤ 0.01.

1.2 Small RNA-seq workflows

1.2.1 Library Preparation

For library preparation, 1 μg of total RNA or 10 ng of enriched small RNA isolated from PTR-EVs was used. The 3′ SR Adaptor for Illumina was ligated to the small RNA using 3′ Ligation Enzyme. To prevent adaptor-dimer formation, excess 3′ SR Adaptor was hybridized with SR RT Primer for Illumina. Subsequently, the 5′ SR Adaptor for Illumina was ligated to the small RNA using 5′ Ligation Enzyme, and first-strand cDNA was synthesized using ProtoScript II Reverse Transcriptase. Each sample was then amplified by PCR using P5 and P7 primers. The PCR products were purified using DNA clean beads, and fragments of 140–160 bp were recovered and cleaned up by polyacrylamide gel electrophoresis (PAGE). Library quality was validated using an Agilent 2100 Bioanalyzer.

Libraries with different indices were multiplexed and sequenced as paired-end reads (PE150) on the Illumina HiSeq X Ten, NovaSeq, or MGI2000 platforms.

1.2.2 Quality Control

Raw sequencing reads in FASTQ format were processed using Trimmomatic (v0.30) to obtain high-quality clean data. Data processing included the following steps:

- Removal of adapter sequences
- Removal of 5′ or 3′ end bases containing Ns or with quality values below Q20
- Trimming of bases with an average quality score below Q20 using a sliding window of 4 bp
- Discarding reads shorter than 18 bp after trimming

1.2.3 miRNA Identification

For fungal species, miRDeep-P2 (v2.1.1.4) was used to identify known and novel miRNAs. All identified miRNAs and their expression data were obtained from the analysis.

1.2.4 Differential Expression Analysis

Differential expression analysis was performed using the DESeq2 Bioconductor package, a model based on the negative binomial distribution. The false discovery rate was controlled using the Benjamini–Hochberg approach. miRNAs with an adjusted p-value < 0.05 and $|\log_2 FC| \geq 1$ were considered differentially expressed.

1.2.5 Target Gene Prediction

Target genes for the identified miRNAs were predicted using TargetFinder (v1.7). Default parameters were applied for target prediction based on sequence complementarity and thermodynamic stability.

1.2.6 GO and KEGG Enrichment Analysis

Gene Ontology enrichment analysis was performed using GOSeq (v1.34.1) to identify GO terms annotating the list of miRNA target genes, with a significance threshold of adjusted p-value < 0.05. The directed acyclic graph was plotted using topGO.

KEGG pathway enrichment analysis was performed using in-house scripts to identify significantly enriched pathways among the target genes. KEGG is a collection of databases dealing with genomes, biological pathways, diseases, drugs, and chemical substances.

1.2.7 Principal Component Analysis

Principal component analysis was performed to reduce data complexity and analyze sample relationships and the scale of differences among samples. The basic principle of PCA is to convert original variables into a new set of independent variables (principal components). All factors are ranked based on significance, with minor factors and noise eliminated to simplify the data.

1.3 RT-qPCR validation

Primers used for RT-qPCR validation of differentially expressed genes (DEGs) were designed using Primer-BLAST. The primer sequences are listed in Table S1. RT-qPCR was performed using the ChamQ SYBR qPCR Master Mix (Without ROX; Q321, Vazyme Biotech Co., Ltd., Nanjing, China). Each 30 μL reaction mixture contained 15 μL of qPCR master mix, 1 μL of cDNA template, 0.6 μL each of forward and reverse primers, and 13.4 μL of ddH_2O. The amplification conditions were as follows: 95 °C for 30 s, followed by cycling at 95 °C for 10 s and 60 °C for 1 min. The 18S gene was used as the internal reference, and relative expression levels were calculated using the $2^{-\Delta\Delta CT}$ method.

Table S1. Primer sequences used for RT-qPCR validation.

Target gene ID	Primers	Sequence (5′→3′)
GME691_g	*GME691_g*-F	ACAAAGCACGATACCGCAGA
	GME691_g-R	ATCCTTGGCGTTCTGCTCAA
GME829_g	*GME829_g*-F	CCATCTCCAGACTACGCTCC
	GME829_g-R	CATGCCGGACATGGGTAGAA
GME8425_g	*GME8425_g*-F	CCAAATCAAAGCACCCAGCC
	GME8425_g-R	AGCAGCGTCCTCCATTGAAA
GME10364_g	*GME10364_g*-F	TCGAACCTTGCAACTCCCTC
	GME10364_g-R	TCTTCTTGGGTGCCAACTCG
GME2725_g	*GME2725_g*-F	CCCGCCTCTTCAAACAGCTA
	GME2725_g-R	CTCACCCGTTGTTTGTGCTG
GME4253_g	*GME4253_g*-F	TGCAAAGAAGACGCAACACG
	GME4253_g-R	AGCATTGACAGGGAACTGGG
GME9069_g	*GME9069_g*-F	CTTGGGCCACATACGACAGT
	GME9069_g-R	ACGCTTGGTCAAAGGGCTTA
GME10337_g	*GME10337_g*-F	TAGTTTCAGGAACGCCCACC
	GME10337_g-R	AGTGGTAAGCGGACTTTGGG
18S	18S-F	TAGAGTCGCCGTAATGAT
	18S-R	GCAGTAGTTGGTCTTGAG

2. Supplementary Characterization and Validation

This section provides supplementary characterization and validation data supporting the selection of *Pleurotus tuber-regium* as the model system and the reliability of the omics analyses. It includes comparative EV characterization across different edible mushrooms, quality assessment of PTR-EV protein preparations, and RT-qPCR validation of selected RNA-seq results. **Figure S1** shows the particle size distribution profiles of EV-enriched fractions isolated from five edible mushroom fermentation broths. These comparative data supported the preliminary evaluation of vesicle-like particle characteristics across species and contributed to the selection of *Pleurotus tuber-regium* as the model system for subsequent analyses.

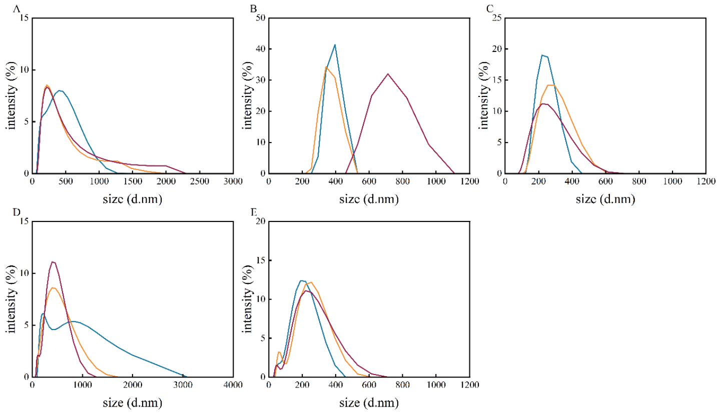

Figure S1. DLS profiles of EVs from different edible mushrooms. (A) *Flammulina velutipes*. (B) *Schizophyllum commune*. (C) *Ganoderma lucidum*. (D) *Cordyceps militaris*. (E) *Pleurotustuber-regium*.

Table S2 summarizes the particle concentrations measured by NTA for EV-enriched fractions obtained from different edible mushroom fermentation broths. The

comparatively higher particle concentration observed for *P. tuber-regium* supported its selection for further characterization and functional evaluation.

Table S2. EV particle concentrations in edible mushroom fermentation broths.

Assignment	Edible mushroom	NTA particle concentration ($\times 10^7$ particles/mL)
A	*F. velutipes*	46.5 ± 1.9
B	*S. commune*	2.0 ± 0.2
C	*G. lucidum*	13.0 ± 0.7
D	*C. militaris*	8.9 ± 0.4
E	*P. tuber-regium*	112.0± 4.2

Figure S2 shows the protein banding pattern of proteins extracted from PTR-EV-enriched fractions. The SDS-PAGE profile was used as a preliminary quality assessment of the protein samples prior to proteomic analysis, indicating the presence of diverse protein components suitable for downstream characterization.

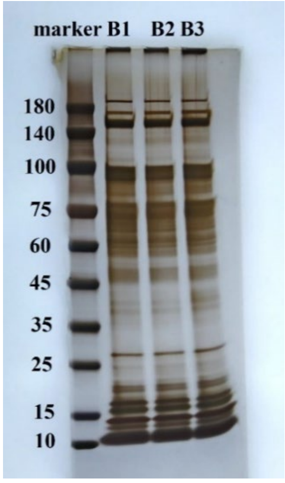

Figure S2. SDS-PAGE analysis of PTR-EV proteins.

Figure S3 shows the KEGG pathway enrichment results for proteins identified in PTR-EVs. The enriched pathways included ribosome, proteasome, oxidative phosphorylation, carbon metabolism, and protein processing in the endoplasmic reticulum. These results suggest that the proteins detected in PTR-EVs are mainly associated with protein synthesis and turnover, energy metabolism, and cellular stress-related processes, thereby providing a functional overview of the PTR-EV proteome.

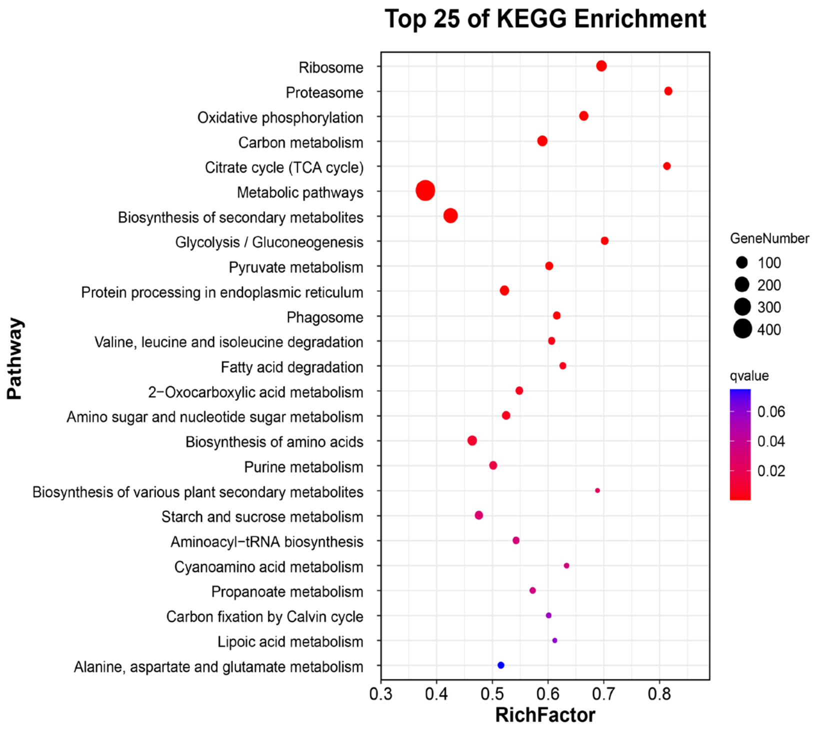

Figure S3. KEGG pathway enrichment analysis of proteins identified in PTR-EVs.

3. Supplementary Extended Omics Results

This section presents extended omics analyses that complement the main manuscript. It includes miRNA target prediction and functional enrichment results, supplementary RNA-seq differential expression and GO enrichment analyses, integrative transcriptome–proteome comparison, and candidate EV-associated biomarker annotation.

3.1 Small RNA sequencing quality assessment and miRNA target analysis

Small RNA sequencing of PTR-EVs generated sufficient depth and high-quality data across all replicates (A1–A3), with Q20 > 98%, Q30 > 95%, low N content, and GC contents of 53–56%, supporting downstream analyses. ncRNA annotation showed that rRNA dominated the small RNA pool, whereas miRNAs were present at low abundance (390–2,841 reads), indicating that miRNAs constitute a minor EV component. Despite this, two novel miRNAs (NovelmiRNA-1 and NovelmiRNA-3) were consistently and highly detected across all samples, with read counts of 860–1,947 (TPM > 2 × 10^5) and 2,565–4,891 (peak TPM ≈ 1 × 10^6), respectively. Both displayed canonical mature lengths (~20 nt), predicted hairpin precursors, and reliable target matching in the PTR genome, supporting their designation as core EV-associated miRNAs (**Table S3**). GO level-2 and enrichment analyses linked their targets to metabolic processes and catalytic activities, with specific enrichment in ubiquitin-related pathways and redox- and receptor-associated functions (**Figure. S4** & **S5**). KEGG annotation identified four targets encoding a ubiquitin-conjugating enzyme (*GME317_g*), an ATP-dependent DNA helicase PIF1 (*GME2987_g*), a polygalacturonase (*GME7569_g*), and an NADH-dependent glutamate synthase (*GME5969_g*) (**Table S4**).

Table S3 summarizes the predicted target genes of the abundant novel miRNAs detected in PTR-EV-enriched fractions, including target matching information and alignment-related parameters. The results provide supplementary evidence supporting the presence of highly represented EV-associated novel miRNAs in PTR-EV-enriched fractions.

Table S3. Predicted target genes of PTR-EV-associated miRNAs.

Seq1	Seq2	Score	Len1	Len2	Positions
NovelmiRNA-3	rna-GME317_g	2.5	20	885	34
NovelmiRNA-3	rna-GME2987_g	4	20	1764	991
NovelmiRNA-1	rna-GME317_g	3.5	20	885	34
NovelmiRNA-1	rna-GME7569_g	4	20	1143	355
NovelmiRNA-1	rna-GME8540_g	4	20	1131	700
NovelmiRNA-1	rna-GME5390_g	4	20	1356	526
NovelmiRNA-4	rna-GME5969_g	3.5	21	6447	938

Figure S4 presents the level-2 GO classification of the predicted target genes of abundant novel miRNAs identified in PTR-EV-enriched fractions. The targets were mainly associated with broad biological functions such as metabolic processes and catalytic activity, providing an overview of their potential functional distribution.

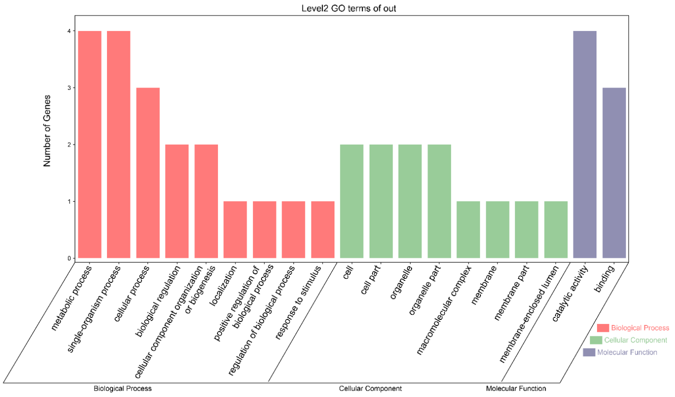

Figure S4. Level-2 GO classification of predicted miRNA target genes.

Figure S5 shows the GO enrichment results for the predicted target genes of abundant novel miRNAs identified in PTR-EV-enriched fractions. The enriched terms suggest potential associations with ubiquitin-related pathways as well as redox- and receptor-associated functions, thereby providing supplementary functional context for the small RNA analysis.

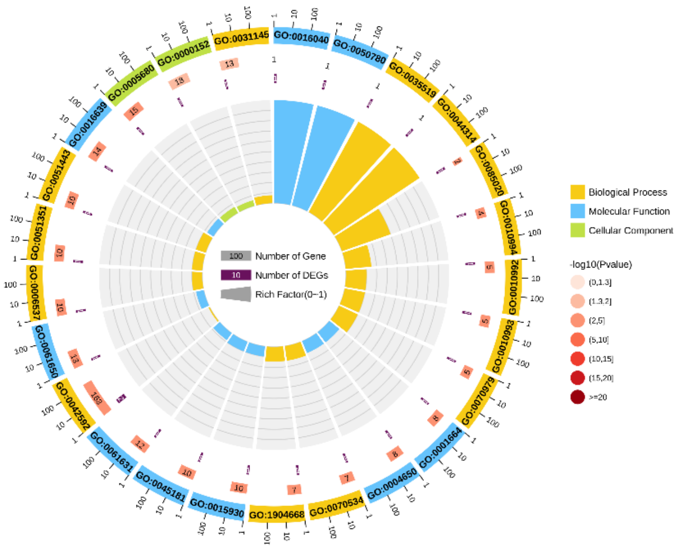

Figure S5. GO enrichment analysis of predicted miRNA target genes.

Table S4 lists representative KEGG pathway annotations for the predicted target genes of abundant novel miRNAs identified in PTR-EV-enriched fractions. Four annotated targets were identified, encoding a ubiquitin-conjugating enzyme (*GME317_g*), an ATP-dependent DNA helicase PIF1 (*GME2987_g*), a polygalacturonase (*GME7569_g*), and an NADH-dependent glutamate synthase (*GME5969_g*), supporting potential links to ubiquitin-mediated proteolysis, DNA-related functions, polysaccharide degradation, and nitrogen metabolism.

Table S4. KEGG pathway annotation of predicted miRNA target genes.

Target gene	KEGG ID	Definition
GME317_g	K10583	UBE2S, E2EPF; ubiquitin-conjugating enzyme E2 S
GME2987_g	K15255	PIF1; ATP-dependent DNA helicase PIF1
GME7569_g	K01184	E3.2.1.15; polygalacturonase
GME5969_g	K00264	GLT1; glutamate synthase (NADH)

3.2 Supplementary transcriptomic and integrative omics analyses

This subsection presents supplementary transcriptomic and integrative omics analyses that complement the main manuscript. It includes the overall distribution of temperature-responsive differentially expressed genes, GO enrichment patterns of upregulated and downregulated genes, the overlap between the PTR-EV proteome and the temperature-responsive transcriptome, and candidate EV-associated biomarkers inferred from KEGG orthology-guided proteomic annotation. Together, these supplementary results provide additional support for the interpretation that temperature stress is associated with coordinated changes in EV production, cargo composition, and stress-related cellular pathways.

Figure S6 presents the level-2 GO term distribution of temperature-responsive differentially expressed genes in PTR. The DEGs were broadly distributed across biological process, cellular component, and molecular function categories, with major terms including cellular process, metabolic process, binding, and catalytic activity. These results provide a general functional overview of the transcriptomic response of PTR to elevated temperature.

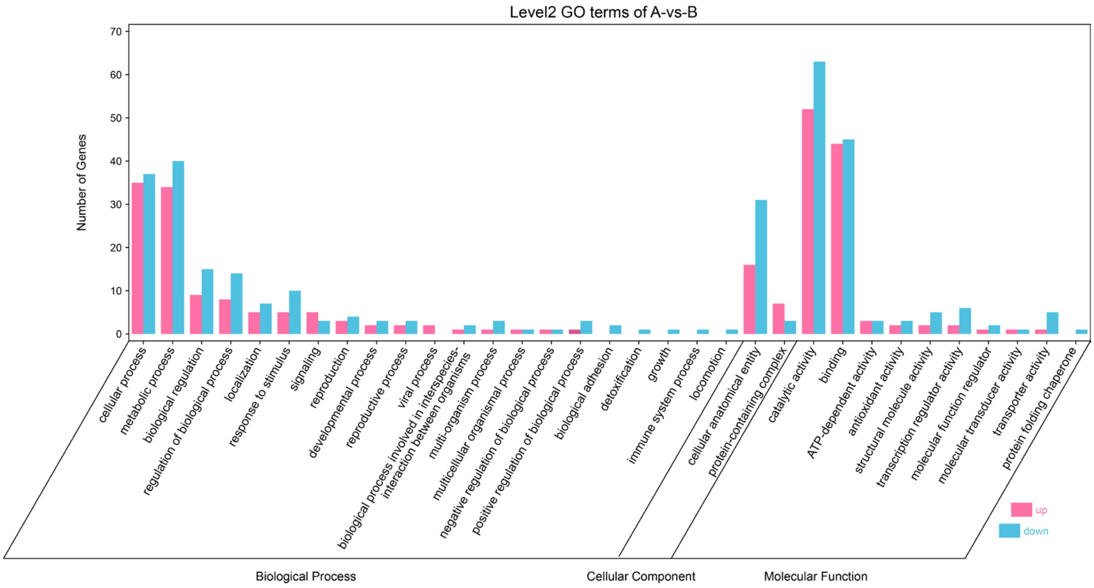

Figure S6. Level-2 GO term distribution of temperature-responsive differentially expressed genes in PTR.

Figure S7 shows the overall distribution of differentially expressed genes identified by RNA-seq under temperature stress. Upregulated and downregulated genes are displayed according to their fold-change and statistical significance, providing a global overview of the transcriptional response associated with altered EV production.

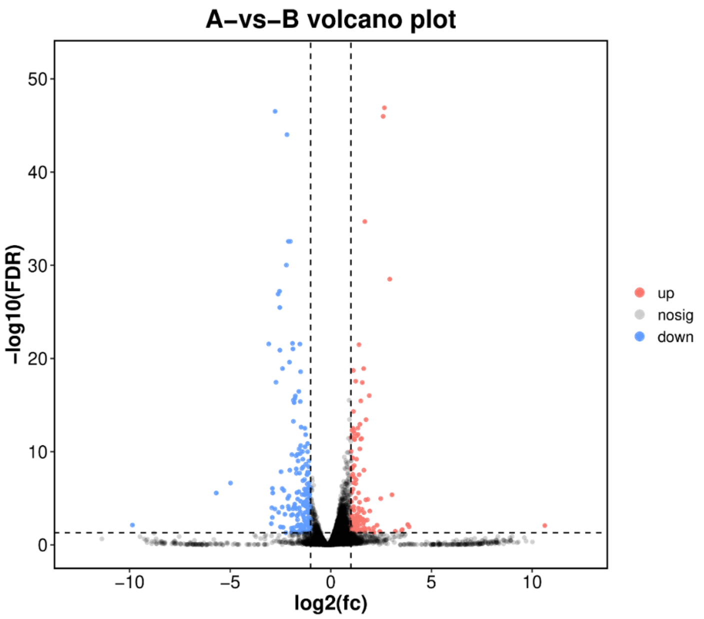

Figure S7. Volcano plot of DEGs identified by RNA-Seq analysis.

Figures S8 and S9 present the top 20 enriched GO terms among downregulated and upregulated genes identified by RNA-seq analysis under temperature stress. Together, they provide supplementary information on the functional categories suppressed or activated under the elevated-temperature condition and complement the transcriptomic interpretation presented in the main manuscript.

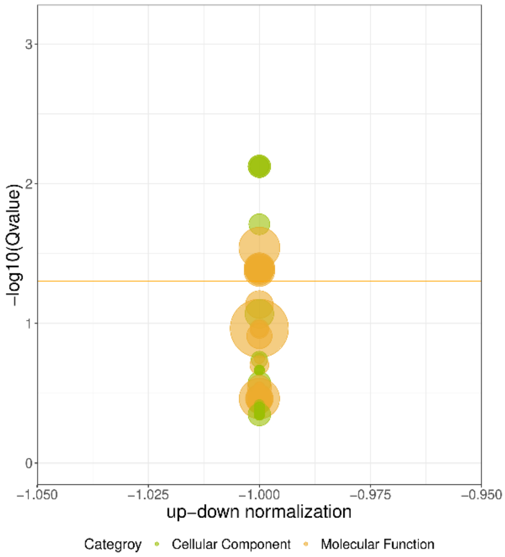

GO term Top 20

ID	Descrption
GO:0005618	cell wall
GO:0042597	periplasmic space
GO:0030312	external encapsulating structure
GO:0009277	fungal–type cell wall
GO:0016491	oxidoreductase activity
GO:0020037	heme binding
GO:0046906	tetrapyrrole binding
GO:0070011	peptidase activity, acting on L–amino acid peptides
GO:0008233	peptidase activity
GO:0008234	cysteine–type peptidase activity
GO:0005506	iron ion binding
GO:0005199	structural constituent of cell wall
GO:0016705	oxidoreductase activity, acting on paired donors, with incorporation or reduction of molecular oxygen
GO:0071944	cell periphery
GO:0003824	catalytic activity
GO:0003995	acyl–CoA dehydrogenase activity
GO:0004601	peroxidase activity
GO:0050660	flavin adenine dinucleotide binding
GO:0003908	methylated–DNA–[protein]–cysteine S–methyltransferase activity
GO:0070069	cytochrome complex

Figure S8. Top 20 enriched GO terms among downregulated DEGs.

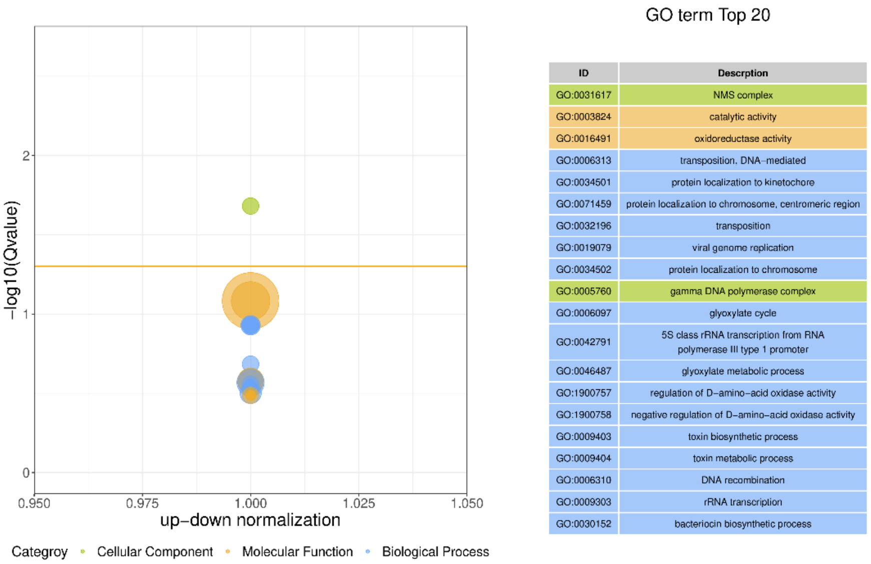

Figure S9. Top 20 GO enriched terms among upregulated DEGs.

Table S5 lists candidate EV-associated biomarkers identified in PTR-EVs through KEGG orthology-guided analysis of the proteomic dataset. The annotated proteins include homologs of EV markers or core components of EV biogenesis pathways reported in other systems, thereby providing supplementary support for EV-associated molecular characterization in PTR.

Table S5. Candidate EV-associated biomarkers identified in PTR-EVs based on KEGG orthology-guided proteomic analysis

Candidate EV-associated protein	KO	Gene ID	Relative quantitative value of protein
HSP40	K09503	*GME6116_g*	532.703796
HSP70	K03283	*GME6174_g*	1709.28748
		GME9194_g	8749.87402
HSP90	K04079	*GME9155_g*	1836.57178
CHMP2	K12191	*GME6121_g*	244.227798
Rab7	K07897	*GME8200_g*	494.587402
VPS4	K12196	*GME6818_g*	227.495087
VPS35	K18468	*GME8428_g*	254.091843

The KEGG pathway annotation was performed on the overlapping genes identified between the proteins in PTR-EVs and the temperature-responsive differentially expressed genes (DEGs). The annotation results are presented in **Table S6**, the full version of which was also provided as a single excel table file. Among these overlapping genes, eight genes were significantly up-regulated and five genes were significantly down-regulated under the 34°C condition.

Table S6. Integrated analysis of PTR EVs proteome and temperature-responsive transcriptome.

Gene ID	Relative quantitative value of protein	Log_2FC of DEGs	KO	Definition
GME7538_g	885.59	1.29	K01638	aceB, glcB; malate synthase
GME6875_g	43349.74	1.00	K02332	POLG; DNA polymerase gamma 1
GME10681_g	37362.76	1.16	K17066	MOX; alcohol oxidase
GME6432_g	175.02	1.07	K13993	HSP20; HSP20 family protein

https://doi.org/10.3390/xxxxx

GME5459_g	123.18	1.24	K19791	FET3_5; iron transport multicopper oxidase
GME6391_g	740.06	1.83	K00273	DAO, aao; D-amino-acid oxidase
GME5194_g	478.19	1.41	K01469	OPLAH, OXP1, oplAH; 5-oxoprolinase (ATP-hydrolysing)
GME10037_g	168.97	2.14	K05857	PLCD; phosphatidylinositol phospholipase C, delta
GME9500_g	107329.75	-1.50	K01309	MINDY1_2; ubiquitin carboxyl-terminal hydrolase MINDY-1/2
GME10336_g	376.20	-1.38	K17862	PPOC; linoleate 10R-lipoxygenase
GME7852_g	7624.06	-1.03	K10355	ACTF; actin, other eukaryote
GME7134_g	244.28	-1.25	K05658	ABCB1, CD243; ATP-binding cassette, subfamily B (MDR/TAP), member 1
GME2725_g	291.24	-1.29	K03885	ndh; NADH:quinone reductase (non-electrogenic)

Figure S10 shows the RT-qPCR validation results for selected differentially expressed genes (DEGs). The expression trends obtained by RT-qPCR were generally consistent with the transcriptomic data for both up-regulated and down-regulated genes, supporting the reliability of the RNA-seq results.

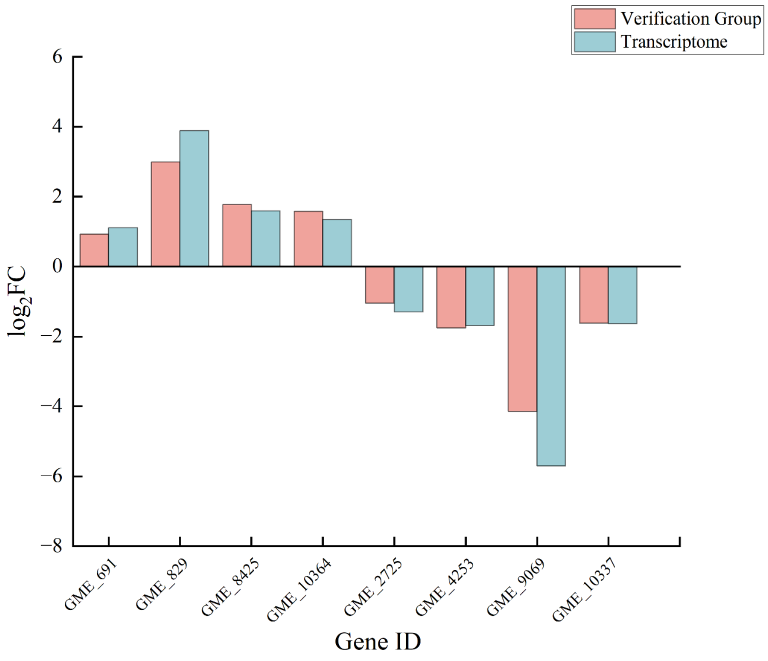

Figure S10. RT-qPCR validation of selected temperature-responsive DEGs.

Disclaimer/Publisher's Note: The statements, opinions and data contained in all publications are solely those of the individual author(s) and contributor(s) and not of MDPI and/or the editor(s). MDPI and/or the editor(s) disclaim responsibility for any injury to people or property resulting from any ideas, methods, instructions or products referred to in the content.
